# Spatiotemporal characteristics of visual cortical responses to transpalpebral electrical stimulation

**DOI:** 10.1016/j.isci.2026.116705

**Published:** 2026-07-10

**Authors:** Meixuan Zhou, Yiheng Xu, Tianyue Meng, Tianruo Guo, Yanyang Zhang, Liqing Di, Liming Li, Heng Li, Xinyu Chai

**Affiliations:** 1 School of Biomedical Engineering, Shanghai Jiao Tong University, Shanghai, China; 2 Department of Physical Education, Shanghai Jiao Tong University, Shanghai, China; 3 School of Biomedical Engineering, University of New South Wales, Sydney, NSW, Australia; 4 Department of Neurosurgery, Chinese PLA General Hospital, Beijing, China; 5 Neurosurgery Institute, Chinese PLA General Hospital, Beijing, China; 6 Department of Orthopedics, Shanghai Pudong Hospital, Shanghai, China

**Keywords:** retinal degeneration, transpalpebral electrical stimulation, visual cortical responses, intrinsic optical signal imaging, electrical stimulation site, computational modeling

## Abstract

Transpalpebral electrical stimulation (TpES) has comparable therapeutic efficacy to transcorneal electrical stimulation (TcES) for retinal neurodegenerative disorders. Characterizing TpES-evoked visual cortical responses is critical to expand the clinical application of minimally invasive neuromodulation. We performed intrinsic optical signal imaging in the cat visual cortex to characterize spatiotemporal neurovascular coupling responses to independent-channel TpES, analyzed retinal electric field distribution via a human head computational model, with TcES as a control in both *in vivo* and simulation experiments. TpES evoked peripheral visual field cortical responses and retinal electric fields consistent with TcES patterns, with similar temporal dynamics. TpES amplitudes were comparable or significantly higher, indicating more efficient visual pathway activation. Our findings provide important evidence supporting the advancement and optimization of non-invasive stimulation techniques for the treatment of retinal neurodegenerative diseases.

## Highlights

• TpES evokes visual cortical responses comparable to TcES in spatiotemporal profile
• TpES response amplitudes indicates greater activation efficiency of visual pathway
• Retinal electric fields shares consistent spatial patterns between TpES and TcES

## Graphical abstract

**Figure:**
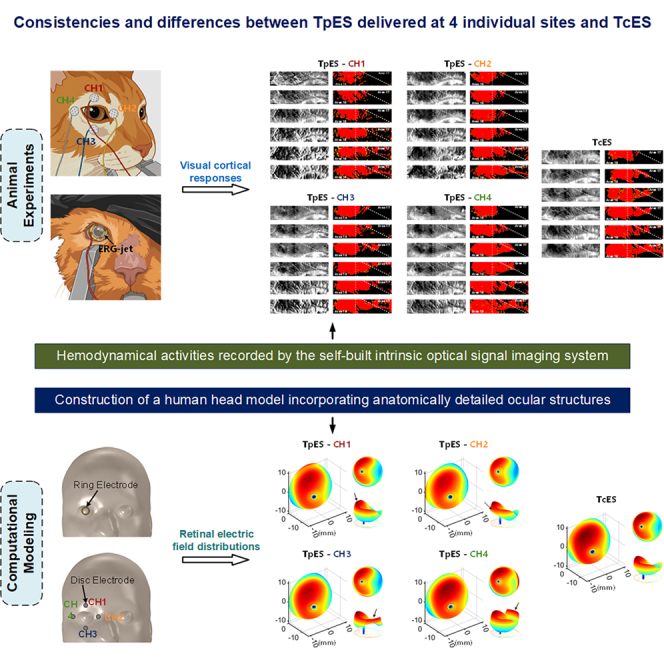

## Teaser

Biological sciences; Feline ophthalmology; Methodology in biological sciences; Sensory neuroscience

## Introduction

Transcorneal electrical stimulation (TcES), supported by well-established neuroprotective mechanisms,^1^^,^^2^ has been clinically confirmed to produce therapeutic benefits in patients with retinal degeneration (RD).^3^^,^^4^^,^^5^ In addition, experimental studies in animal models have demonstrated that TcES enhances retinal cell survival,^6^^,^^7^^,^^8^ and elicits strong neural responses throughout the visual pathway, including both the retina and the visual cortex.^9^^,^^10^^,^^11^ Despite these advantages, the stimulation electrodes used in TcES, such as Dawson-Trick-Litzkow (DTL)-Plus or electroretinography (ERG)-jet electrodes, are placed directly on the corneal surface.^12^^,^^13^ This contact-based configuration has been associated with adverse effects in some patients, including ocular discomfort, dry eye syndrome, and pain during RD treatment.^14^^,^^15^ Furthermore, the restoration of visual function typically requires multiple sessions of electrical stimulation delivered over repeated treatment courses.^16^^,^^17^ Consequently, although TcES is considered minimally invasive, the discomfort associated with the procedure may reduce patient compliance and limit their willingness to complete standardized multi-course therapy.

Transpalpebral electrical stimulation (TpES), which delivers microcurrents through electrodes placed on the eyelid skin, has also demonstrated therapeutic potential in the treatment of ocular disorders, with no treatment-related adverse events reported to date.^18^^,^^19^^,^^20^^,^^21^ Furthermore, a previous *in vivo* study supported by immunofluorescence analysis showed that TpES does not produce detectable damage to the ocular surface. In contrast, TcES was found to directly disrupt mucin homeostasis within the corneal tear film.^22^ This multilayered tear film plays a crucial role in maintaining optical refraction and preserving the epithelial barrier function of the ocular surface.^23^^,^^24^ Although TpES appears to offer clear safety advantages over TcES, the direct functional relationship between TpES and coordinated neural activation within the visual pathway has not yet been clearly defined. In comparison, such functional associations have already been systematically established for TcES.^9^^,^^10^ The absence of this evidence represents a significant knowledge gap that limits the comprehensive evaluation of TpES for clinical use. In particular, the neural responses induced by TpES have not been systematically characterized. These responses represent an important indicator of the functional integrity of the retinocortical pathway,^25^ and it remains uncertain whether TpES can elicit neural activity patterns comparable to those produced by TcES. Additionally, most previous TpES studies have employed stimulating electrodes that cover part or the entirety of the palpebral skin.^19^^,^^20^^,^^26^ A related investigation demonstrated that the location of stimulation can alter the distribution of current density across the retina.^27^ However, it is still unclear whether differences in stimulation sites influence the spatiotemporal characteristics of the responses evoked along the visual pathway.

Given the well-established retinotopic mapping between the retina and the primary visual cortex,^28^^,^^29^^,^^30^ electrophysiological and optical measurements reflecting cortical neural activity have been extensively used to characterize retinal responses to external stimulation.^31^^,^^32^^,^^33^ This approach provides a reliable and validated framework for utilizing cortical activity to investigate the neurophysiological effects evoked by TpES. In parallel, computational modeling of retinal electric field distributions has become a widely accepted methodological approach in TcES-related studies for evaluating and optimizing electrical stimulation strategies.^34^^,^^35^ Based on these considerations, the primary objective of the present study was to systematically investigate the spatial, temporal, and amplitude characteristics of TpES-induced hemodynamic responses at multiple discrete palpebral stimulation sites. This was achieved using intrinsic optical signal (IOS) imaging of the visual cortex in an *in vivo* adult cat model. To complement the neurophysiological experiments, additional simulations were conducted to analyze the spatial distribution of electric fields generated by stimulation at each TpES site. These simulations employed a multi-conductivity human head model incorporating detailed anatomical structures of the eye. TcES was used as a parallel control condition in both the *in vivo* IOS imaging experiments and the retinal electric field simulations.

## Results

### Spatial properties of visual cortical responses evoked by TpES

**Figure 1:**
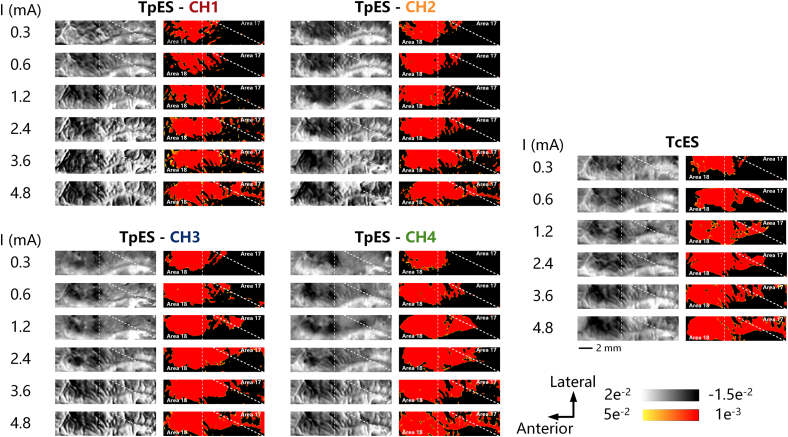
Spatial distribution of visual cortical responses Left column of each panel: single-condition functional map within the cortical field of view; right column: corresponding pixel-wise *p* value map (*p* < 0.05, one-tailed *t* test). The grayscale bar indicates pixel grayscale values, and the color bar shows one-tailed *t*-test *p*-values for the corresponding pixels.

Within the cortical region exposed for IOS imaging, Figure 1 illustrates the single-condition functional map exhibiting the strongest response and its corresponding pseudo-color map, highlighting pixels that were significantly activated (*p* < 0.05, one-tailed *t* test). Stimulation was delivered using biphasic rectangular pulses at current intensities ranging from 0.3 to 4.8 mA. Cortical responses evoked by all TpES channels displayed similar spatial distribution patterns, which closely resembled those elicited by TcES. At relatively low stimulation intensities (<1.2 mA), TpES-CH1 through -CH4 and TcES induced significant activation primarily within a subregion of Area 18, corresponding topographically to the peripheral visual field in the cat.^36^ As the stimulus current increased, the activated cortical region expanded progressively, extending into Area 17, which represents the central visual field.^37^

**Figure 2:**
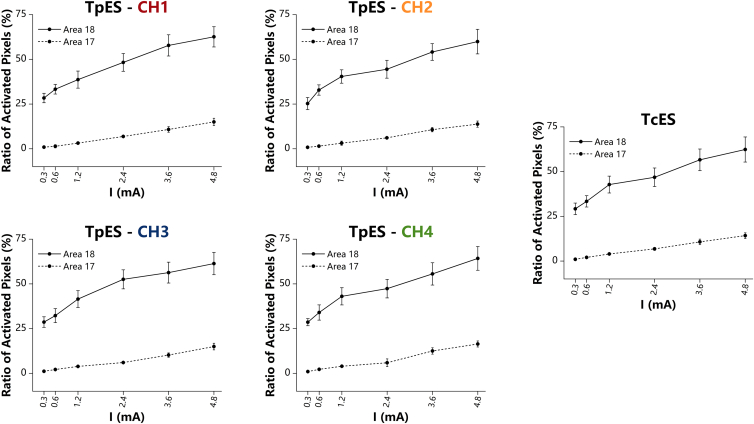
Quantitative characterization of the spatial properties of visual cortical activation Each panel shows the ratio of significantly activated pixels in the peripheral (Area 18, solid lines) and central (area 17, dashed lines) visual cortex evoked by TpES-CH1 to -CH4 and TcES. Data points and error bars indicate mean ± SEM (*n* = 11).

Figure 2 illustrates a similar trend in the proportion of activated pixels across Areas 18 and 17: The fraction of significantly activated cortical regions, as shown in Figure 1, increased with stimulation current in both areas. Notably, the spatial extent of hemodynamic responses was consistently larger in the peripheral visual cortex (Area 18) compared to the central region (Area 17), regardless of whether stimulation was delivered via any TpES channel or by TcES. Table S1 summarizes the percentages of significantly activated cortex evoked by TpES and TcES across all tested current intensities. At each intensity, the proportion of activated cortex in both Areas 17 and 18 was comparable across all four TpES channels and the TcES control condition. To formally evaluate the effect of stimulation site, a one-way analysis of variance (ANOVA) was performed for each current intensity with stimulation site as the between-subject factor (five levels: TpES-CH1 through CH4, TcES; *n* = 11 per group). Across all current intensities, these analyses revealed no significant main effect of stimulation site on the percentage of activated visual cortical area (all *p* > 0.05).

### Temporal features of visual cortical responses evoked by TpES

**Figure 3:**
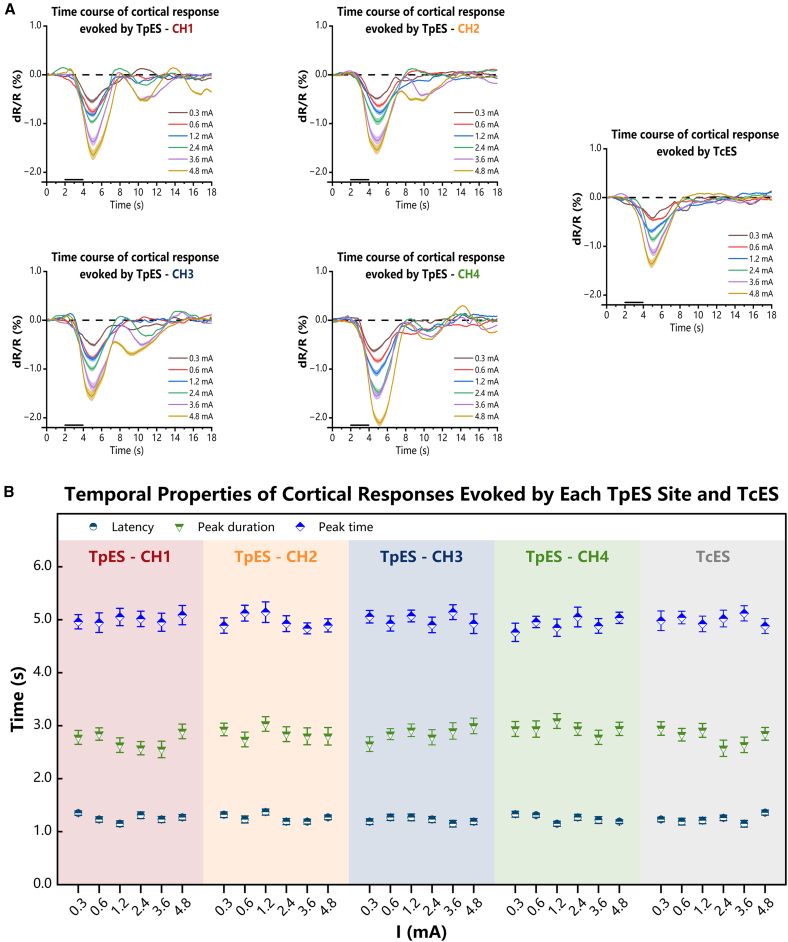
Temporal characteristics of visual cortical responses to TpES and TcES (A) Time courses of the mean evoked response in the selected ROI. Each trace shows the mean ± SEM hemodynamic response (*n* = 11) across 0.3 to 4.8 mA stimulation currents. The horizontal dashed line in each panel indicates the baseline of the evoked cortical response, and the black horizontal bar on the bottom left denotes the duration of the electrical stimulation. (B) Quantitative analysis of the temporal features of the evoked responses. Response latency (navy circles), peak time (blue diamonds), and peak duration (green triangles) were extracted from the averaged time courses of the selected ROI. Data points and error bars represent the mean ± SEM across all 11 animals, respectively.

Time courses derived from the mean gray value of the selected region of interest (ROI), which exhibited the strongest response amplitude, are shown in Figure 3A. Visual cortical responses evoked by all TpES channels and the TcES control displayed similar temporal profiles. Specifically, the absolute dR/R increased rapidly immediately after stimulation onset, reached its peak within 1–2 s, and then gradually returned to baseline over the subsequent 10 s. Figure S1 presents the sequential dR/R maps evoked by TpES and TcES at a fixed current intensity (3.6 mA), further confirming these temporal characteristics. Despite the overall similarity, peak amplitudes varied across stimulation sites. For example, at 4.8 mA, TpES-CH4 elicited a larger peak response than the other TpES channels and TcES (yellow curves in Figure 3A). Figure 3B quantitatively summarizes the temporal parameters of visual cortical responses in the selected ROI for all TpES channels and TcES. All hemodynamic signals exhibited an onset latency of approximately 1.2–1.3 s relative to baseline dR/R onset (TpES-CH1: 1.23 ± 0.057 s; TpES-CH2: 1.19 ± 0.047 s; TpES-CH3: 1.15 ± 0.061 s; TpES-CH4: 1.22 ± 0.063 s; TcES: 1.15 ± 0.064 s; I = 3.6 mA, *n* = 11). Response peaks occurred around 5 s (TpES-CH1: 4.95 ± 0.171 s; TpES-CH2: 4.84 ± 0.104 s; TpES-CH3: 5.14 ± 0.140 s; TpES-CH4: 4.88 ± 0.139 s; TcES: 5.12 ± 0.143 s; I = 3.6 mA, *n* = 11), consistent with the minimum values in Figure 3A. Response durations exceeded 2.5 s for all conditions (TpES-CH1: 2.55 ± 0.155 s; TpES-CH2: 2.80 ± 0.155 s; TpES-CH3: 2.90 ± 0.155 s; TpES-CH4: 2.78 ± 0.134 s; TcES: 2.64 ± 0.145 s; I = 3.6 mA, *n* = 11). Although subtle differences in temporal parameters were observed across conditions, two sets of one-way ANOVAs revealed no statistically significant main effects (all *p* > 0.05). Separate analyses across each of the six tested current intensities (five stimulation sites: TpES-CH1 to CH4, TcES; *n* = 11; F(4, 40)) detected no significant effect of stimulation site. Similarly, tests performed across each of the five stimulation sites (six current intensities; *n* = 11; F(5, 50)) revealed no significant effect of current intensity.

### Amplitude characteristics of visual cortical responses evoked by TpES

**Figure 4:**
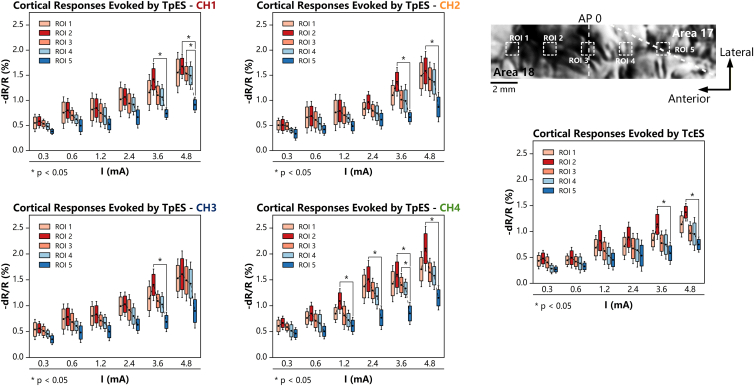
Averaged peak cortical response amplitudes evoked by TpES channels and TcES across predefined regions of interest (ROIs 1 to 5) Top right inset: Localization of ROIs 1 to 5 on the single-condition functional map. Black dots = mean, boxes = mean ± SEM (*n* = 11), whiskers = mean ±1.5 × SEM. ∗*p* < 0.05: significant pairwise differences from Tukey’s HSD post hoc test, performed only after a significant main effect in one-way ANOVA.

Figure 4 presents the peak amplitudes of cortical responses evoked by the four individual TpES sites and the TcES control condition, calculated from the mean gray values of the five predefined ROIs (top-right inset of Figure 4) in the single-condition functional maps. Across all stimulation conditions, response amplitudes in ROIs 1–5 increased progressively with rising current intensity. Notably, ROI 2, located within Area 18, consistently exhibited the strongest cortical activation for both TpES and TcES. Response amplitudes decreased anteriorly toward ROI 1 and posteriorly from ROIs 3 to 5, with the lowest response observed in ROI 5, which partially encompasses Area 17.

Remarkably, at a fixed current intensity, separate one-way ANOVAs revealed a significant main effect of ROI (five levels: ROI 1-ROI 5) on peak cortical response amplitude for both TpES-CH1 and TpES-CH4 (TpES-CH1: F(4, 40) = 4.722, *p* < 0.05, I = 4.8 mA; TpES-CH4: F(4, 40) = 3.567, *p* < 0.05, I = 3.6 mA; *n* = 11). Post hoc analyses using Tukey’s Honestly Significant Difference (HSD) test indicated that, for TpES-CH1, responses in ROIs 2–3 were significantly stronger than those in ROI 5 (*p* = 0.016, 0.027, respectively), whereas for TpES-CH4, responses in ROIs 2–3 were significantly higher than those in ROI 5 (*p* = 0.033, 0.041, respectively). The significant differences in response amplitude across different ROIs are also evident in the visual cortical hemodynamic signals evoked by other TpES channels (TpES-CH2, TpES-CH3) and TcES at a given current intensity, as shown in Figure 4. This spatial pattern, where peripheral cortical regions (typically ROIs 2–3, corresponding to Area 18) exhibited stronger evoked response amplitudes than the central cortical region (ROI 5, corresponding to Area 17), was consistently observed across other stimulation intensities, as well as for the remaining TpES channels and the TcES control condition.

**Figure 5:**
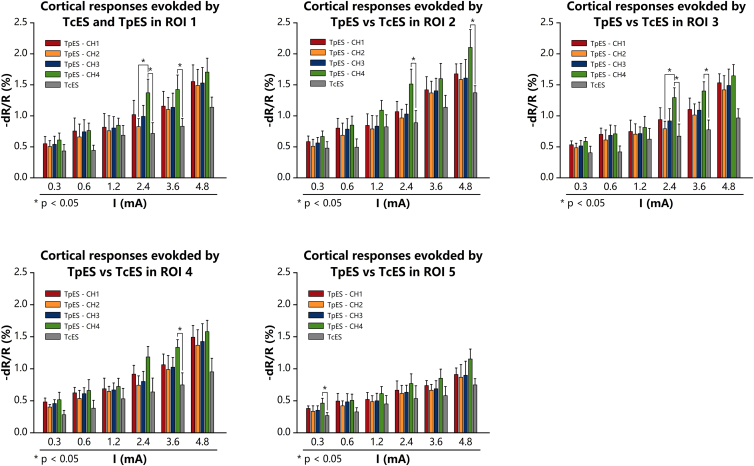
Comparison of cortical response amplitudes evoked by TpES channels and TcES across 5 predefined cortical ROIs Red, orange, blue, green, gray bars: mean response amplitude across all animal subjects (*n* = 11) for TpES-CH1, CH2, CH3, CH4, and TcES, respectively. Bar height = mean, error bars = mean ± SEM. ∗*p* < 0.05: significant pairwise differences from Tukey’s HSD post hoc test, performed only after a significant main effect of stimulation condition in one-way ANOVA.

To further examine the amplitude characteristics of cortical responses evoked by the four individual TpES sites and the TcES control within the same cortical region, we reorganized the data from Figure 4 from a stimulation site-based layout into an ROI-based presentation. Figure 5 shows that TpES-CH4 consistently elicited the highest peak amplitudes across all predefined ROIs. TpES-CH1 and TpES-CH3 produced comparable levels of cortical activation, whereas TpES-CH2 evoked weaker responses relative to the other TpES sites. The TcES control consistently elicited the lowest visual cortical activation across all five selected ROIs. Statistical analysis revealed that stimulation site or paradigm significantly influenced cortical response amplitudes across ROIs (one-way ANOVA; five groups: TpES-CH1 to CH4 and TcES). Significant main effects were observed at the following conditions: ROI 1 at 2.4 and 3.6 mA; ROI 2 at 2.4 and 4.8 mA; ROI 3 at 2.4 and 3.6 mA; ROI 4 at 3.6 mA; ROI 5 at 0.3 mA (*p* < 0.05). Post hoc analyses using Tukey’s HSD test indicated that these effects were primarily driven by TpES-CH4 eliciting significantly stronger cortical responses than TcES across all ROIs at the specified intensities (ROI 1: I = 2.4, 3.6 mA, *p* = 0.033, 0.044; ROI 2: I = 2.4, 4.8 mA, *p* = 0.028, 0.033; ROI 3: I = 2.4, 3.6 mA, *p* = 0.015, 0.028; ROI 4: I = 3.6 mA, *p* = 0.020; ROI 5: I = 0.3 mA, *p* = 0.042). Additionally, TpES-CH4 also induced significantly greater activation than TpES-CH2 in ROI 1 and ROI 3 when I = 2.4 mA (*p* = 0.029, 0.041).

### Spatial distributions of retinal electric fields

**Figure 6:**
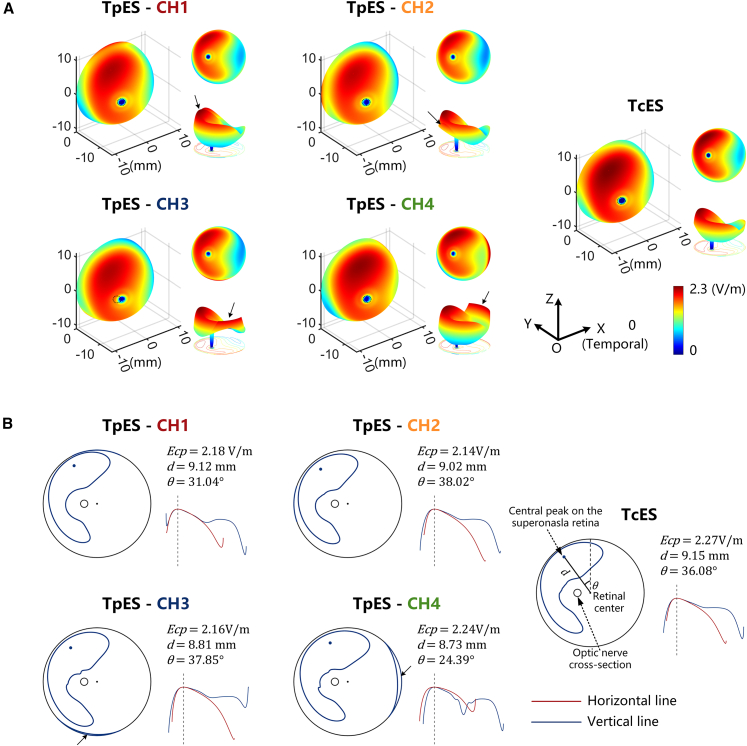
Spatial characteristics of retinal electric fields generated by four individual TpES channels and TcES (A) Electric field distributions across the posterior retina for each stimulation paradigm. Each inset includes a 3D view (main panel), an XOZ cross-sectional projection (upper right), and a 3D mesh plot (lower right) of the retinal electric field. Color bar indicates the absolute electric field amplitudes on the retinal surface. (B) 80% intensity contours of the within-condition min-max normalized electric field for each TpES channel and TcES. Each inset shows the 80% contour (dark blue line, main panel), Ecp and central peak position (upper right), and distribution curves of electric field with the Ecp as the maximum, extending bilaterally along the horizontal (red) and vertical (blue) directions. Solid arrows: site-specific enhancements of electric fields and 80% contours from individual TpES channels at the corresponding retinal edge.

Figure 6A illustrates the spatial patterns of normalized electric fields generated by the four individual TpES channels and the TcES control. Across all conditions, retinal electric field distributions were broadly similar, with each stimulation paradigm producing a widespread, diffuse field over the posterior retina. Specifically, the position of the peak field was conserved across conditions, consistently localizing to the superonasal retina. The peak intensity of all electric fields exceeded 2 V/m, and it decreased gradually in all radial directions from this peak, becoming negligible near the optic nerve head. Subtle variations were observed in the XOZ projections (upper-right of each inset) and 3D meshes (lower-right of each inset) of the retinal electric fields. These differences were characterized by locally enhanced electric fields associated with each TpES channel, corresponding to the anatomical position of its stimulating electrode, rather than the TcES control. For example, TpES-CH4 induced relatively high-amplitude fields along the temporal edges of the posterior retina.

Figure 6B further presents a quantitative characterization of the electric field distributions. All 80% maximum intensity contours exhibited a “crescent-shaped” pattern along the nasal aspect of the posterior retina, consistently sparing the optic nerve head region, and tapering progressively from the superior to inferior retinal edge. The central electric field intensity at the peak (Ecp) generated by TpES-CH1 to -CH4 and TcES in the superonasal retina was 2.18 V/m, 2.14 V/m, 2.16 V/m, 2.24 V/m and 2.27 V/m, respectively. Taking this peak center as the origin, the horizontal and vertical distribution profiles of retinal electric fields (lower-right of each inset) further confirm a gradual attenuation trend of electric field intensity from the superonasal region to the surrounding retina. Notably, secondary branches of the 80% intensity contour emerged along the temporal retinal edge for TpES-CH4, spatially aligned with the electrode location, whereas this feature was nearly absent in the contours generated by other TpES channels and TcES. Differences in the central peak position were also detected across stimulation paradigms. The distances (d) between the peak and the posterior retinal geometric center were nearly identical (∼9 mm) for all conditions, whereas the angular eccentricities (θ) varied: highest for TpES-CH2, followed by TpES-CH3, TcES, TpES-CH1, and lowest for TpES-CH4.

## Discussion

This study provides neurophysiological evidence via IOS imaging, that TpES delivered at discrete palpebral sites reliably elicits robust visual cortical responses. *In vivo* recordings demonstrated that these responses exhibit spatiotemporal profiles comparable to those evoked by the TcES control, while also revealing site-dependent differences in response amplitude across specific visual cortical ROIs. Complementary simulations using a multi-conductivity human head model confirmed a conserved superonasal peak in the retinal electric field across all stimulation paradigms, with localized field enhancements corresponding to the anatomical positions of individual TpES electrodes.

Together, these findings establish a functional framework for investigating the TpES-evoked activation of the visual pathway and the underlying cortical circuitry, supported by biophysical insights from computational modeling. The results suggest that non-invasive TpES represents a promising strategy for retinal electrical stimulation in early- to mid-stage RD, with the rational selection of stimulation sites serving as an important consideration for optimizing therapeutic efficacy.

### TpES evoked consistent visual cortical responses with TcES and existing studies

The visual pathway of cats shares many structural and functional similarities with that of humans, including ocular dimensions, retinal cell distribution, and cortical representation of the visual field,^38^^,^^39^^,^^40^ making cats a widely used model for investigating retinal, optic nerve, and visual cortical responses to electrical stimulation.^10^^,^^11^^,^^41^ In the present study, TpES-evoked visual cortical responses were consistent with the well-established TcES-evoked response profiles reported in previous work.

**Figure 7:**
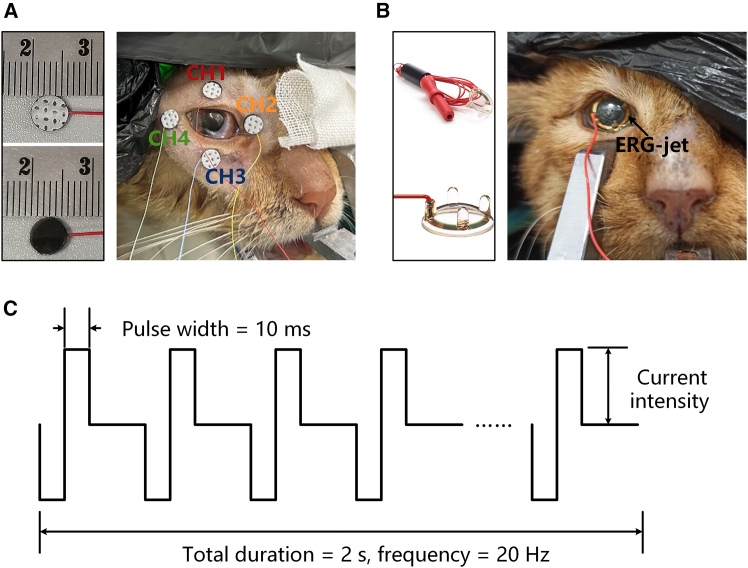
Schematic of animal experiments (A) Custom-modified, self-adhesive skin electrode and montage of four individual TpES sites. TpES channels CH1 to CH4 were placed on the superior, nasal, inferior, and temporal palpebral skin, respectively. (B) Schematic of the commercial ERG-jet electrode and its placement on the corneal surface for TcES. (C) Biphasic charge-balanced, cathode-first rectangular pulse waveform and corresponding stimulation parameters delivered by TpES and TcES.

First, as shown in Figures 1 and 7A, TpES applied to the superior, nasal, inferior, and temporal palpebral surfaces consistently elicited cortical activity prominently localized to Area 18, with spread into Area 17 observed at higher current intensities. This spatiotemporal activation profile was not only comparable to the TcES control (Figure 7B) in our study but also aligned with prior investigations of TcES parameter optimization using ∼530 nm green-light IOS imaging, which have demonstrated that the ERG-jet electrode preferentially activates ganglion cells in the peripheral retina rather than those in the central retina.^9^^,^^11^^,^^42^ In the cat visual system, Area 18 is topographically specialized for processing peripheral visual field inputs, whereas Area 17 primarily encodes the central visual field.^36^^,^^37^ This topographic organization explains why cortical responses detected by IOS imaging were predominantly localized to Area 18 at low stimulus intensities and extended toward Area 17 as stimulus current increased. With our observation of similar spatial distributions of visual cortical response (Figures 1 and 2), it was suggested that TpES and TcES might share a common mechanism characterized by diffuse and preferential activation of the peripheral retina.

**Figure 8:**
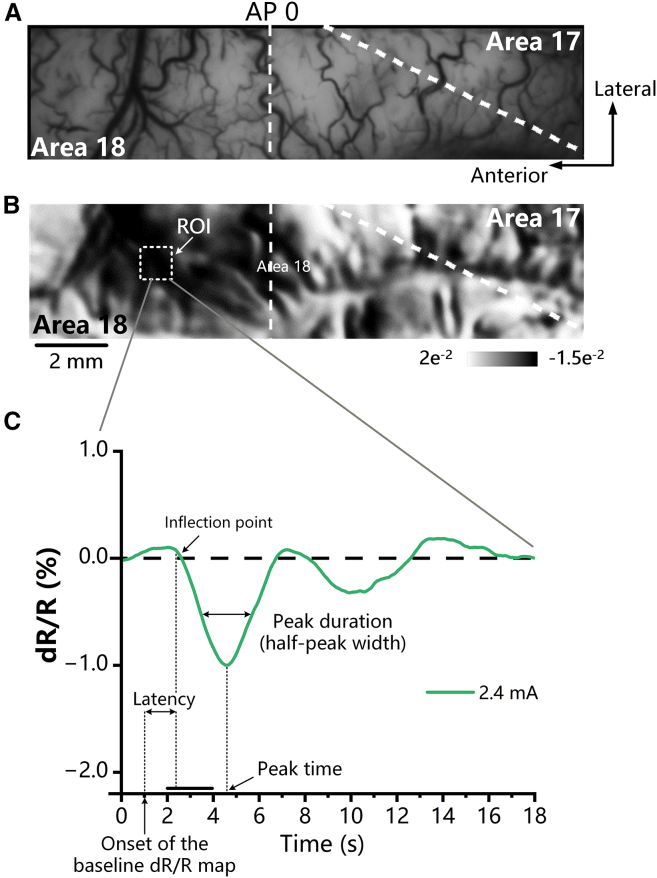
Cortical exposure, ROI illustration, and temporal response analysis (A) Cortical exposure under IOS imaging, overlaid with the AP0 reference in the Horsley-Clarke coordinate system and the boundary between Areas 17 and 18 delineated from cortical responses evoked by drifting grating stimulation. (B) A typical single-condition functional map with the identified 1 mm^2^ ROI exhibiting the strongest response amplitude. (C) Temporal response parameters were derived from the mean pixel time course (bottom) within a 1 mm^2^ ROI showing the strongest activation.

Second, the temporal profiles and characteristic parameters of neurovascular coupling signals evoked by TpES channels and TcES (Figures 3 and 8) were also consistent with existing literature. Specifically, the response latency (∼1.3 s relative to baseline dR/R) matched previous reports for TcES-evoked responses using rectangular or sinusoidal pulses.^43^ The peak response occurred around the third second after stimulation onset (fifth second in the present study), and the response duration exceeded 2.5 s, consistent with temporal properties recorded in IOS studies of the retina and visual cortex.^11^^,^^43^ However, given the limited temporal resolution of our IOS system (25 ms per frame), higher-resolution approaches, such as *in vivo* cortical electrophysiology or direct measurements of retinal neural and vascular activity,^44^^,^^45^ will be required to fully characterize TpES-evoked visual pathway responses.

Although our findings on TpES- and TcES-evoked cortical activation are consistent with previous reports, our analytical focus differs from prior studies that systematically characterized the spatial distribution and intensity dependence of TcES-evoked negative responses in Area 17, often interpreted as inhibitory signals potentially reflecting shifts in the balance of excitatory and inhibitory neuronal activity.^46^^,^^47^ The primary goal of the present study was to validate the feasibility of TpES and to characterize the excitatory visual cortical responses, which directly reflect retinal neuron activation and are linked to the neuroprotective mechanisms underlying the therapeutic efficacy of retinal electrical stimulation.^1^^,^^2^ Accordingly, we focused on the robust excitatory cortical responses and did not perform a systematic analysis of the synchronous inhibitory signals observed in Area 17, consistent with the emphasis of prior IOS studies.^11^^,^^42^ A comprehensive investigation of TpES-evoked inhibitory signals will be a priority in our future mechanistic studies.

### Peripheral retinal activation and site specificity of TpES

**Figure 9:**
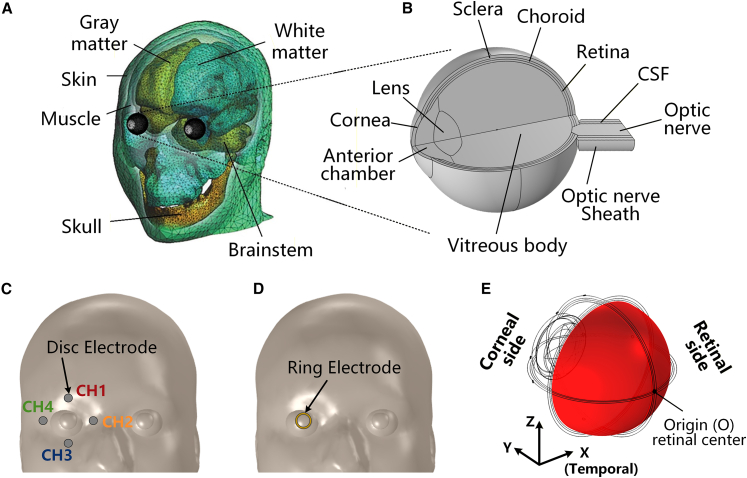
Schematic of computational modeling (A) Human head model (left) embedded with a detailed eye model (right). Reproduced with permission from Cvetković et al.^80^ (CC BY). (B) Magnified view of the eye model incorporating anatomically realistic tissue structures. (C and D) Montages of the stimulating electrodes for four individual TpES channels and TcES. A common return electrode was fixed at the back of the neck and was not illustrated here. (E) Retinal surface for the analysis of electric field distribution in Figure 6.

Across all conditions, Figures 1 and 2 show that both TpES applied at four independent stimulation sites and the TcES control evoked substantially stronger hemodynamic responses in visual cortical Area 18 compared to Area 17. Beyond this inter-area difference, we observed notable heterogeneity in response amplitude across the analyzed ROIs within Area 18 (Figure 4). In particular, ROI 2, anatomically localized to the anterior segment of Area 18 in the hemisphere contralateral to the stimulated eye, was identified pixel-wise based on the single-condition functional map exhibiting the strongest cortical activation. This ROI consistently exhibited higher response amplitudes than the other Area 18 ROIs (ROIs 1, 3, and 4), with amplitudes significantly exceeding those of ROI 5 in Area 17. The peak activation in ROI 2 further indicates that both TpES and TcES preferentially drive maximal activation in the superonasal retinal subregion topographically mapped to the inferior temporal visual field.^36^^,^^37^ Moreover, the progressive decline in cortical response amplitude from ROI 2 to anterior ROI 1 and posterior ROIs 3–5 reflects the spatial attenuation of retinal neuronal activation from the superonasal central peak toward surrounding regions, such as the central retina. This peripheral activation pattern is further corroborated by our computational modeling on the head model with detailed ocular structures established in Figure 9,which revealed that TpES and TcES generate highly consistent electric fields across the posterior retina, peaking in the superonasal periphery and decaying gradually toward adjacent regions (Figure 6). Notably, under a simulated current of 1 mA, the central peak intensity (Ecp) of the retinal electric field induced by all TpES channels and TcES in the superonasal retinal region approximates 2 V/m. This magnitude falls within the same order of magnitude as the peak electric field strength (0.2–2 V/m) on the retinal surface reported in previous studies with analogous electrode configurations.^48^^,^^49^^,^^50^ Electric fields of this magnitude are capable of exerting therapeutic effects via the entrainment of rhythmic firing of retinal ganglion cells (RGCs).^51^^,^^52^

Previous in silico and *in vivo* studies using the same stimulation paradigm as in the present work (with the return electrode fixed at the posterior neck) have shown that TcES generates electric fields peaking in the nasal region of the peripheral retina and evokes hemodynamic responses with maximal activation localized to the relatively anterior portion of Area 18 in the visual cortex contralateral to the stimulated eye.^11^^,^^34^^,^^43^ Moreover, clinical investigations have demonstrated that electrical stimulation delivered through ERG-jet and self-adhesive electrodes both induce conscious phosphenes in the temporal visual field,^12^^,^^53^ which topographically corresponds to the nasal retinal region in humans.^54^ Together, these findings suggest that TpES applied at different palpebral sites and TcES via an ERG-jet electrode share a common visual pathway activation pattern: Both produce diffuse retinal electric fields with a specific peripheral peak, which in turn elicit hemodynamic responses in the visual cortex that decrease progressively in amplitude from the strongest activation in the peripheral region to surrounding cortical areas.

In addition, our results reveal the site specificity of TpES-evoked visual cortical response amplitudes relative to stimulating electrode placement. As shown in Figure 5, the response amplitudes elicited by the four individual TpES channels varied across all analyzed visual cortical ROIs, with statistically significant differences between specific conditions. This phenomenon can be attributed to site-specific electric field enhancement at the peripheral retinal edges, spatially aligned with the location of the stimulating electrode in TpES paradigms, but absent in the TcES control (top-right of each inset in Figure 6A). Notably, TpES-CH4 produced the most pronounced localized electric field enhancement at the temporal retinal edge, with an amplitude approaching that of the superonasal central peak. The 80% intensity contour in Figure 6B further demonstrates that TpES-CH4 can theoretically recruit retinal neurons at the temporal edge when the electric field intensity reaches the activation threshold in this region.^55^ Given the functional connectivity of the visual pathway,^56^ this additional temporal edge activation superimposes on the main input from the superonasal retinal peak, ultimately producing the stronger cortical response amplitude observed for TpES-CH4. In contrast, this spatial feature of the retinal electric field is far less prominent for the other TpES-channels (e.g., TpES-CH2) and is virtually undetectable in the TcES condition.

The site-specific differences in retinal electric field may stem from variations in the anterior segment of the eye, consistent with preclinical evidence that the TpES stimulation site can modulate retinal current density.^27^ In single-channel paradigms, the electric field peak is typically concentrated near the stimulating electrode.^57^^,^^58^ Consequently, the distinct electrode positions of the TpES channels and TcES produce divergent electric field distributions within anterior ocular tissues, in contrast to the highly consistent field observed across the posterior retina (Figure 6). Nevertheless, the primary aim of our computational modeling was to characterize the posterior retinal electric field, which is justified for two reasons: (1) neuronal activation in this region underlies the therapeutic effects of retinal electrical stimulation for RD and related disorders^49^; and (2) it provides a complementary explanation for the evoked visual cortical responses in terms of retinal topography. While our simulations do not capture the anterior ocular field distribution, it is reasonable to hypothesize that high-intensity fields in the anterior segment propagate along distinct pathways to the peripheral retinal edges, driving localized enhancement of retinal network activation. This augmented input is then transmitted through the visual pathway,^59^ ultimately contributing to the stronger visual cortical hemodynamic responses observed with TpES, particularly TpES-CH4 (Figure 5).

Although the visual systems of cats and humans share extensive structural and functional similarities in ocular axial length, retinal ganglion cell receptive fields, and topographic organization of the visual cortex, the cat’s retina lacks the primate-specific fovea.^40^ Distinct interspecific differences also exist in the spatial distribution of photoreceptors and ganglion cells, as well as in intra- and inter-retinal neural connectivity.^60^^,^^61^ These interspecific disparities indicate that hemodynamic responses evoked in the feline visual cortex by TpES and TcES at different eyelid locations cannot be directly extrapolated to human phosphene perception. Nevertheless, adopting human head computational models to interpret experimental results is scientifically justified. Firstly, human-based modeling helps clarify the fundamental mechanisms of retinal activation, laying a theoretical foundation for TpES clinical application.^49^^,^^53^ Secondly, cross-species computational studies demonstrate that anatomical and conductivity differences do not significantly alter electric field spatial characteristics.^62^^,^^63^ Validated anatomical and conductivity parameters in human simulation models also ensure reliable simulation results.^34^^,^^35^ Furthermore, our model enables quantitative evaluation of retinal electric field distribution and intensity, parameters that are difficult to measure *in vivo*. This facilitates the interpretation of spatiotemporal properties in visual cortical responses observed in cat experiments. We acknowledge interspecific differences in ocular properties, such as the feline nictitating membrane. With a thickness of 10–100 μm and limited spatial extent relative to the eye and stimulation electrodes, it causes only minor, localized perturbations to the electric field near the medial canthus, with negligible impact on global electric fields and deep structures such as the retina.^64^ Differences in cranial morphology and size mainly alter the location of retinal electric field peaks via electrode positioning, rather than changing overall field distribution or activation patterns.^65^ Therefore, future studies combining feline cranial and ocular-based electric field simulations with the preclinical evaluation of TpES site effects on phosphene perception will provide robust evidence for the site-specific activation of the peripheral retina and visual cortex induced by TpES.

### TpES possesses potential advantages to TcES in retinal electrical stimulation

Our study is the first to demonstrate that visual cortical responses evoked by TpES, delivered via electrodes positioned on the superior, nasal, inferior, and temporal palpebral surfaces, exhibit spatial and temporal profiles comparable to those elicited by TcES using an ERG-jet electrode (Figures 1, 2, 3, and 4). Importantly, TpES consistently induced hemodynamic responses with greater amplitude than TcES in specific cortical regions (Figure 5). These results suggest that TpES may provide an advantage over TcES in terms of visual pathway activation efficiency, while retaining its well-established noninvasive benefits.^19^^,^^20^^,^^22^

An underlying reason for this phenomenon may arise from variations in current conduction pathways.^22^ To address this issue, we performed a preliminary qualitative assessment of current streams generated in ocular and cranial tissues by the four independent TpES sites and TcES, as illustrated in Figure S2. Consistent with the comparable spatial electric field distributions across the retinal surface for TpES and TcES (Figure 6A), the spatial patterns of current streams throughout the cranial and ocular tissues also exhibit overall consistency across all four TpES channels and TcES, with only subtle differences in the anterior eye, particularly in the periorbital region. These differences are characterized by two main features: First, TcES-generated current streams passing through the retina can penetrate the anterior eye; second, TpES generates an additional current component that “circumvents” the eyeball via the orbital margin to reach the retinal surface, as indicated by red arrows for TpES-CH1 and -CH3 in Figure S2A, and TpES-CH4 in Figure S2B. The expected nasal-side bypass current for TpES-CH2 was not observed in Figure S2, likely due to the limited mesh segmentation resolution of our head model. The third row of Table S2 further demonstrates that TpES generates a higher electric field in the peripheral retinal edges (outside the 80% intensity contours), particularly for TpES-CH4, with a maximum value of 2.11 V/m compared to 1.63 V/m for TcES. This site-specific current component drives local enhancement of the electric field at the retinal edges aligned with the TpES electrode positions (Figure 6A), resulting in shifts of the central peak of the retinal electric field across the four TpES sites (top-right corner of each inset in Figure 6B). Such variations plausibly account for the differences in amplitude observed in evoked cortical responses, consistent with the integrative nature of visual cortical signals, which combine afferent input from all ascending visual pathways along with subcortical horizontal and vertical interconnections.^59^ Although the retinotopy of the IOS imaging area maps to the nasal retina in the cat,^36^^,^^37^ the stronger cortical responses evoked by TpES (Figure 5) may be attributed to these site-specific enhancements of the retinal electric field relative to TcES.

However, the first two rows of Table S2 indicate that the maximal electric field intensity in the superonasal (Ecp) and in the central retina (a region centered on the macula with a diameter of 5.5 mm),^65^ generated by TcES, is comparable to or higher than that of TpES channels. Therefore, the site-specific enhancement of electric fields at the peripheral retinal edge cannot be regarded as the exclusive factor for the stronger visual cortical responses evoked by TpES. Alternatively, another potential influencing mechanism may stem from differences in impedance, including resistive components, between the stimulation sites and the palpebral skin or corneal surface during dynamic electrical stimulation. Theoretically, the stimulation area of the ERG-jet electrode is approximately 20.6 mm^2^, comparable to or slightly larger than the single self-adhesive electrode used for TpES (19.6 mm^2^),^66^ which implies that TcES delivers a relatively lower current density and is less invasive.^67^ However, Yang et al. demonstrate that TcES can damage corneal epithelial cells compared with TpES even when applied below the nominal safety threshold. Furthermore, the electrical resistance between the electrode site and the retina after electrical stimulation is four times higher than that of TpES.^22^ Notably, the cornea is a multilayered structure composed of the epithelial layer, Bowman’s layer, and stroma. Some of these layers cannot regenerate after mechanical or electrical damage and are instead replaced by newly formed scar tissue fibers, which may alter the electrical conductivity between the stimulation sites and retinal target regions.^68^^,^^69^ The electrical resistance between the electrode site and the retina after electrical stimulation can be four times higher than that of TpES. Additionally, the crystalline lens possesses an intrinsically high electrical resistance,^70^ which suggests that TcES currents should traverse this high-impedance structure, whereas TpES provides a more efficient pathway to reach the retina through periocular skin with high-conductivity vasculature.^71^

Impedance and resistance at the electrode-tissue interface are dynamic and can change during the course of electrical stimulation. First, the steady-state electrostatic field simulations employed in our computational modeling do not capture these electrophysiological fluctuations. Second, the *in vivo* IOS imaging experiments required repeated trials and blocks at varying stimulation intensities to characterize the spatiotemporal properties of visual cortical responses and enhance the signal-to-noise ratio of intrinsic signals. This design precluded real-time monitoring of dynamic changes in the electrical properties of the electrode-tissue interface, as well as their dependence on specific stimulation parameters. Future studies could address this limitation by systematically characterizing these dynamic interface properties using *in vivo* neurophysiological recordings,^72^ employing stimulation paradigms that elicit robust visual pathway responses.

An alternative hypothesis posits that TpES at high stimulation intensities (4.8 mA) may induce the widespread polarization of intracranial tissues, potentially activating the visual cortex via a non-retinal pathway, analogous to transcranial electrical stimulation,^73^ while Table S2 also confirms that TpES (∼0.9 V) has higher electrode potentials relative to TcES (0.68 V). However, as shown in Figure S3, under the simulated current of 1 mA, all TpES channels and TcES generated consistent patterns of electric field in the whole brain, with relatively strong electric field intensities in the prefrontal cortex adjacent to the stimulation electrodes and eyeballs, as well as in the brainstem near the return electrodes. The maximum electric field of the whole cerebral cortex ranges from 0.83 to 1.11 V/m under various stimulating sites (fifth row of Table S2). Additionally, as shown in the last row of Table S2, TpES-CH1 to -CH4 and TcES exhibit peak values of 0.24, 0.23, 0.21, 0.18 and 0.15 V/m, respectively, in the peri-visual cortex outlined by the dashed box in Figure S3. It can be anticipated that as stimulation current intensity increases, the electric fields generated by all TpES sites and TcES near the visual cortex can reach or exceed the threshold of 0.2–0.8 V/m for direct neuronal activation.^74^^,^^75^ However, the hemodynamic responses (Figures 1, 2, 3, 4, and 5) recorded in the cat visual cortex may not stem from the direct stimulation of the imaged visual cortex by TpES or TcES. First, Figures 1, 2, and 4 display region- and amplitude-specific differences in evoked responses between visual cortical Areas 18 and 17, which are consistent with the selective activation of Y-type and X-type RGCs in the peripheral and central retina, respectively.^76^^,^^77^ This degree of spatially selective cortical activation cannot be achieved through single-channel transcranial electrical stimulation, due to current shunting through superficial tissues.^78^ Secondly, the temporal characteristics of visual cortex responses evoked by all TpES channels and TcES at various stimulation intensities are identical (Figure 3), exhibiting a distinct signal pattern initiated by retinal neuron activation and transmitted to the visual cortex through the visual pathway.^9^^,^^79^ Besides, our IOS imaging approach was limited to Areas 17/18 of the cat’s anterodorsal visual cortex.^36^^,^^37^ Under high stimulation intensities, TcES and TpES may directly activate the more posterior Areas 17/19 adjacent to the return electrode at the back of the neck,^36^ with neural signals propagating to the exposed imaging region via subcortical horizontal conduction pathways, where the temporal characteristics of subcortical signal transmission exceed the temporal resolution limit of the IOS imaging system.^59^ Since the head model adopted in this study lacks refined the segmentation of cortical subregions such as the primary visual cortex (V1),^80^ accurate localization of maximal electric fields near the visual cortex is limited (Figure S3). Accordingly, future studies should employ high-resolution head models with detailed cortical segmentation to achieve accurate electric field localization in the visual cortex.^48^ Meanwhile, *in vivo* electrophysiological recordings should also be performed across a larger visual cortical region to analyze the spatiotemporal characteristics of signals in distinct areas.^45^ Furthermore, targeted pharmacological blockade of retinal synaptic transmission can be applied to distinguish retinal and direct cortical contributions to TpES- or TcES-evoked responses.^79^

### Clinical translation considerations for TpES

In clinical practice, when microcurrents are delivered to the retina via TcES or TpES, the return electrode is commonly positioned on the temple,^12^^,^^15^^,^^20^ arm,^81^ or forehead.^4^ computational modeling studies have similarly placed the return electrode on the top of the head or behind the ears.^82^^,^^83^ To maintain methodological consistency with existing *in vivo* TcES studies,^11^^,^^43^ however, we fixed the return electrode at the back of the neck for both our IOS imaging experiments and human head model simulations. Previous work has demonstrated that the return electrode montage can significantly influence the spatial distribution and modulation of retinal electric fields generated by conventional single-channel TcES or temporally interfering (TI) stimulation strategies.^34^^,^^55^^,^^65^ Therefore, in future translational studies, return electrode placement should be guided by clinical applications.^15^^,^^20^ Notably, we hypothesize that the substantial distance between the stimulating and return electrodes, nearly an order of magnitude greater than the eyeball diameter, may contribute to the highly consistent retinal electric field distributions observed across all individual TpES channels and TcES in Figure 6A. Integrating these findings with electrode optimization algorithms will be essential to explore stimulation paradigms capable of achieving targeted retinal current density distributions,^27^^,^^49^ thereby enabling personalized therapeutic strategies tailored to lesion topography in retinal diseases.

It is also important to note that in clinical applications, both TpES and TcES typically involve a single stimulating electrode fixed at a specific site, such as covering the entire palpebra,^20^^,^^26^ or placed on the corneal surface.^12^^,^^15^ Our results demonstrated that four discrete TpES sites consistently evoked visual cortical responses with higher amplitudes than TcES in specific regions (Figure 5), particularly TpES-CH4, where the electrode was positioned on the temporal palpebra. These findings suggest that the phosphene threshold, a critical parameter for determining stimulation current in clinical RD treatment,^3^^,^^4^^,^^84^ may be lower for TpES than for TcES. An unresolved question remains whether the phosphene threshold for TpES delivered via an electrode covering the entire palpebra aligns with that observed using our single 5-mm disc electrode approach.^20^^,^^26^ A further consideration is whether the higher current density resulting from the smaller electrode area, which is a critical factor for electrical stimulation safety,^67^ maintains the safety profile reported in current TpES studies.^19^^,^^20^

Additionally, simultaneous TpES delivery using multiple palpebral electrodes at a fixed total current represents a promising avenue for clinical translation. This strategy could enhance safety by reducing the charge injection density of each individual electrode.^67^ Previous multi-channel neuromodulation studies using electric field crosstalk or TI paradigms have laid the theoretical groundwork for multi-channel TpES, enabling non-invasive, targeted modulation of retinal activation patterns.^35^^,^^55^^,^^57^ However, two critical aspects of multi-channel TpES remain unvalidated: whether visual pathway responses elicited by multi-electrode stimulation remain consistent with single-channel TpES in a manner dependent on total applied current, and the extent to which multi-channel TpES can modulate retinal electric field distributions and visual cortical responses, including its spatial resolution and effective stimulation range.

### Limitations of the study

A primary limitation of this study is that the key findings were derived from *in vivo* animal experiments, which constrains direct clinical translation. For example, it remains unclear whether the spatiotemporal characteristics of TpES-evoked cortical responses correspond to those elicited by TcES delivered via a DTL-Plus electrode, a clinically common approach that can induce ocular side effects.^14^^,^^15^ In addition, the phosphene thresholds established in clinical studies may differ from the stimulation current amplitudes used here to evoke robust cortical responses,^14^^,^^85^ and it is unknown whether TpES can generate visual perceptions comparable to TcES at clinically relevant intensities, which are typically in the tens to hundreds of microamperes.^3^^,^^84^ Therefore, future investigations should employ human head models incorporating detailed ocular structures, adopt clinically standardized stimulating and return electrode configurations, and examine neuronal responses across retinal regions evoked by each TpES modality in accordance with established protocols.^35^^,^^86^

A second important limitation concerns our computational model. Although ocular structures were segmented at the micrometer scale,^43^^,^^65^ head tissues reconstructed from MRI data retained only millimeter-level spatial resolution, and the palpebral skin was not modeled as a separate tissue compartment.^80^^,^^87^ These anatomical simplifications may introduce systematic deviations in the simulated current distribution. Therefore, future high-resolution ocular models should incorporate tissues known to influence TcES- and TpES-induced retinal electric fields, including the palpebral skin, ciliary muscle, and zonules of Zinn.^88^ Another methodological limitation is that retinal conductivity values (Table S3) were derived from averaged published data. Given the heterogeneous distribution of retinal neurons,^89^ and the distinct electrochemical properties of photoreceptors, bipolar cells, and ganglion cells across the multilayered retina,^86^ it will be essential to establish the relationship between retinal conductivity and neuronal density, refine the retina into a physiologically realistic multi-conductivity structure, and integrate more detailed cranial and ocular anatomy in future modeling efforts.

## Resource availability

### Lead contact

Requests for further information and resources should be directed to and will be fulfilled by the lead contact, Dr. Heng Li (liheng@sjtu.edu.cn).

### Materials availability

This study did not generate new unique reagents.

### Data and code availability

• All data reported in this paper will be shared by the lead contact upon request.
• The code used for IOS imaging and analysis, as well as retinal electric fields analysis, is available in Zenodo at https://doi.org/10.5281/zenodo.20730362.
• Any additional information required to reanalyze the data reported in this paper is available from the lead contact upon request.

## Acknowledgments

We sincerely thank Professor Mario Cvetkovié, who shared the raw data of the head model for this research. This study was supported by the 10.13039/501100012166National Key Research and Development Program (grant no. 2025YFF0518002) of China, the 10.13039/501100001809National Natural Science Foundation of China (grant nos. 62073221, 62103269, 62371294), the Natural Science Foundation of Shanghai (grant no. 25ZR1401181), the New Faculty Initiation Program of 10.13039/501100004921Shanghai Jiao Tong University (grant no. 23X010501996), 10.13039/501100001773University of New South Wales (UNSW) 3Rs Fund (RG245361), and the 10.13039/100013805Retina Australia Research Grants (RG253022).

## Author contributions

Conceptualization, X.C. and H.L.; methodology, M.Z., Y.X., T.M., and Y.Z.; investigation, M.Z., Y.X., and L.D.; writing – original draft, M.Z.; writing – review and editing, T.G., L.L., and H.L.; funding acquisition, T.G., L.L., H.L., and X.C.; resources, H.L. and X.C.; supervision, H.L. and X.C.

## Declaration of interests

The authors declare no competing interests.

## Declaration of generative AI and AI-assisted technologies in the writing process

During the preparation of this work, the author(s) used DeepSeek and Doubao (ByteDance) in order to check spelling and grammar. After using this tool or service, the author(s) reviewed and edited the content as needed and take(s) full responsibility for the content of the publication.

## STAR★Methods

### Key resources table

**Table:** 

REAGENT or RESOURCE	SOURCE	IDENTIFIER
**Experimental models: Organisms/strains**		

Adult domestic cat	Tairi Yingen Laboratory Animal Research Facility, Fengxian, Shanghai, China	N/A

**Software and algorithms**		

MATLAB	https://ch.mathworks.com/products/matlab.html	version 2024b
Python	https://www.python.org/	version 3.10
Origin	https://www.originlab.com/index.aspx?go=PRODUCTS/Origin	version 2025
COMSOL Multiphysics	https://cn.comsol.com/comsol-multiphysics	version 5.4
SimNIBS	https://simnibs.github.io/simnibs/	version 4.1
3D Slicer	https://download.slicer.org/	version 5.10.0
Geomagic Wrap	https://support.geomagic.com/s/article/Geomagic-Wrap	version 2024
Custom code	Zenodo: https://doi.org/10.5281/zenodo.20730362	N/A

**Other**		

Tiletamine and zolazepam hydrochloride	https://cn.virbac.com/	Zoletil 50
Isoflurane	https://www.rwdls.com/	0.5%
Tropicamide ophthalmic solution	https://www.bausch.com/	6 mL: 30 mg

### Experimental model and study participant details

Eleven sexually mature adult domestic cats with a mean body weight of 2.91 ± 0.57 kg were purchased from Tairi Yingen Laboratory Animal Research Facility (Fengxian, Shanghai, China). This cohort contained both male and female individuals; sex was not considered as a grouping factor during animal allocation and subsequent data analysis. All animals were individually ventilated cages at the Experimental Animal Center of Shanghai Jiao Tong University under controlled environmental conditions: constant temperature 26 °C, humidity 40-60%, and a 12-hour light/dark cycle, with free access to granule cat food and sterile water. All animal studies were conducted according to the National Institutes of Health (NIH) Principles of Laboratory Animal Care and the ethical guidelines of Shanghai Jiao Tong University (approval number: BME.Ethics.2020012).

The primary readouts of this work are visual cortical responses evoked by retinal electrical stimulation. This study was not designed to investigate sex-based differences, so no sex-stratified subgroup analysis was performed. We acknowledge that subtle sex-related effects cannot be fully excluded under the current experimental design, which constitutes a minor limitation of this study.

### Method details

#### Animal preparation

The surgical procedures have been previously described in detail.^11^^,^^43^ Briefly, eleven adult cats were initially anesthetized by intramuscular injection of tiletamine and zolazepam hydrochloride (0.1 ml/kg). Anesthesia was subsequently maintained using artificial ventilation with isoflurane (2%-3%). The animals were then positioned in a stereotaxic frame (SN-3N, Narishige, Japan), and a craniotomy was performed in the left hemisphere according to Horsley-Clarke coordinates to expose visual areas 17 and 18 (AP -8 to -10 mm; ML -0.5 to -6 mm), contralateral to the stimulated eye. Afterward, a stainless-steel chamber with a diameter of 26 mm was fixed onto the skull using dental cement, and the dura mater covering the exposed cortex was carefully removed. The chamber was then filled with warm dimethylpolysiloxane and sealed with a glass coverslip to minimize motion artifacts and maintain stable intracranial pressure.

Tropicamide ophthalmic solution (0.5%) was administered to induce pupillary dilation in the experimental animals. A flexible corneal contact lens was then placed on the right eye to maintain corneal hydration. In addition, a stainless-steel eyelid retractor was used to prevent the palpebral skin and nictitating membrane from obstructing the corneal surface. Following these preparations, the concentration of isoflurane was reduced to 0.5%-1% and maintained at this level for the remainder of the experiment. The depth of anesthesia was monitored using end-tidal carbon dioxide (ETCO2) measurements displayed on a portable multiparameter monitor. This system allowed continuous real-time monitoring of several physiological parameters, including respiratory rate, electrocardiogram (ECG), body temperature, and ETCO2.

#### Intrinsic optical signal (IOS) imaging system

The IOS imaging system was constructed and controlled using custom-written Python software. The primary hardware setup included a CMOS camera (pco.panda 4.2bi, PCO AG, Germany) equipped with a compatible lens (FA2520A, CHIOPT, China) mounted above the cranial chamber, and a circular LED light source (RIN-120-6R-30G, Oriental Vision System Ltd, Singapore) powered by a programmable DC-stabilized supply (DP832, RIGOL, China) to provide uniform illumination of the visual cortex. A multifunctional device (USB-6002, National Instruments, USA) was used to synchronize the stimulation and imaging recordings. For this study, a 523 nm green light was selected. At this wavelength, oxyhemoglobin (HbO) and deoxyhemoglobin (HbR) in the cortical capillaries have equal absorption coefficients, corresponding to the isosbestic point.^90^ Under these conditions, the recorded IOS predominantly reflects changes in cerebral blood volume (CBV) associated with neuronal activity.^91^ Moreover, CBV signals measured at the isosbestic point exhibit higher amplitudes and improved signal-to-noise ratios (SNR) compared to baseline.^42^ The camera was configured to acquire images at 40 frames per second, with the focal plane positioned approximately 500 μm below the cortical surface. Each trial lasted 18 seconds, comprising a 2-second pre-stimulation baseline, 2 seconds of concurrent stimulation and imaging, and a 14-second post-stimulation period. All images were cropped to include only the cortical region of interest.

#### Visual grating stimulation and boundary delineation of visual cortical areas 17 and 18

All experiments were conducted in a completely darkened room, with the left eye of each cat occluded. Before applying TpES or TcES, visual grating stimulation was delivered to the right eye. This procedure served two purposes: to confirm the functional integrity of the visual pathway in each experimental animal and to delineate the boundary between visual cortical Areas 17/18.^36^^,^^37^

After a 30-minute dark adaptation, the experimental animal was fixed 28.5 cm ahead of an LED monitor, and the area centralis of its right eye was aligned to the center of the screen using a fundus reflective projection.^92^ Full-screen sinusoidal-wave stimulation with drifting gratings at 0.14 and 0.58 cycles per degree (cpd) spatial frequency (SF) were generated on the LED monitor through customized Python program. Each block had 16 trials, in which 0° and 90° drifting gratings (as shown in the middle inset of Figure S4) were given in an interleaved fashion. Each trial contained a 2-second pre-stimulation baseline, 2 seconds of concurrent stimulation and imaging, and a 14-second post-stimulation period. For each SF condition, 8 blocks were performed in total.

The paradigms for delineating the boundary between visual cortical Areas 17 and 18 were adapted from Su et al.,^43^ and detailed in Figure S4. We first computed the Euclidean distance between baseline and post-stimulus time-binned dR/R maps. Five consecutive image frames (0.5 s total duration) with the peak response (maximum Euclidean distance) were selected for averaging. All peak dR/R maps from the same SF condition were averaged across all blocks to generate orthogonal orientation (0° and 90°) single-condition functional maps. As previously reported, Area 18 neurons are preferentially tuned to low-SF gratings (0.14 cpd), whereas Area 17 neurons favor high-SF stimuli with 0.58 cpd.^93^^,^^94^ The functional boundary between Areas 17 and 18 was thus delineated by subtracting the high-SF single-condition functional map from the low-SF map,^9^ an approach validated by prior electrophysiological and histological studies to reliably localize the Area 17/18 boundary.^94^

#### Transpalpebral and transcorneal electrical stimulation

As shown in Figure 7A, four disk-shaped electrodes, each approximately 5 mm in diameter and adapted from self-adhesive skin electrodes composed of hydrogel, conductive carbon film, and medical non-woven fabric (Suwu, Suzhou Kangzhiyun Medical Technology Co., Ltd.) were used for TpES.^95^ Prior to electrode placement, the fur surrounding the palpebral region of the cat’s right eye was fully shaved and cleaned. Each electrode was then individually attached to the superior (CH1), nasal (CH2), inferior (CH3), and temporal (CH4) sides of the palpebral skin, serving as independent TpES stimulation sites. The electrodes were positioned approximately 2 cm from the center of the eyeball. For TcES, the flexible corneal contact lens was replaced with a commercially available ERG-jet electrode, and 0.9% saline was applied to the corneal surface to maintain hydration, as illustrated in Figure 7B. A stainless-steel electrode was inserted into the neck muscle to serve as a common return electrode, consistent with previous TcES animal experiments.^9^^,^^11^

The microcurrent stimulation paradigms used for each TpES site and for TcES are illustrated in Figure 7C. Biphasic, charge-balanced, cathode-first rectangular pulses were generated using a microcurrent stimulator (STG 4004, Multi-Channel Systems, Germany). Each phase had a pulse width of 10 ms, and the pulse frequency was set to 20 Hz, consistent with parameters commonly employed in clinical retinal electrical stimulation studies.^5^^,^^15^^,^^96^ Stimulation was delivered for 2 seconds per trial, as in previous literature, and six current intensities (0.3, 0.6, 1.2, 2.4, 3.6, and 4.8 mA) were selected to fall within the range reported in prior TcES studies.^11^^,^^43^

Electrical stimulation was first delivered through TpES-CH1 at intensities of 0.3, 0.6, 1.2, 2.4, 3.6, and 4.8 mA. Each intensity comprised four blocks, with each block containing 16 trials. Subsequently, stimulation was applied sequentially via TpES-CH2, TpES-CH3, TpES-CH4, and TcES, with four blocks administered at each of the six stimulation intensities. Finally, the stimulation sites were tested in reverse order, TcES, TpES-CH4, TpES-CH3, TpES-CH2, and TpES-CH1, again delivering four blocks at each intensity.

#### Data analysis

All data were processed using custom Python programs. First, the raw images were converted into light reflectance change maps (dR/R) by calculating the relative change in reflectance compared to a baseline image. The baseline image (R_baseline) was generated by binning the initial 1-s frames. This procedure, expressed in “dR/R = (R - R_baseline)/R_baseline”, served to eliminate slow biological noise and correct for uneven illumination across the imaging field. In this analysis, R represented each frame within the total of 720 frames. For each block, the dR/R maps were averaged on a trial-by-trial basis and subsequently binned into 100-millisecond frames for further processing. The boundary between visual cortical areas 17 and 18, as well as the AP0 reference point in the Horsley-Clarke coordinate system (shown in Figure 8A), was determined using the analytical procedures displayed by Figure S4. Further details regarding the methods can be found in the supplemental information.

We analyzed the spatial distributions of visual cortical responses evoked by the four individual single-channel TpES sites and TcES, following the framework illustrated in Figure S5 based on the framework described by Su et al.^43^ For each stimulation condition, dR/R maps from the corresponding time windows across all trial blocks were averaged. The Euclidean distance between the baseline dR/R map and the post-stimulation time-binned dR/R maps was then calculated.^43^ To construct the single-condition functional maps, five consecutive dR/R frames encompassing the peak response window (total duration 0.5 s) were averaged. To reduce low-frequency physiological noise arising from cardiac and respiratory activity, the maps were smoothed using a 7 × 7-pixel Gaussian kernel. A one-tailed *t* test was applied to each single-condition functional map to identify pixels showing a significant decrease in reflectance (*p* < 0.05) relative to the baseline.^97^ These pixels were subsequently encoded in pseudo-color to generate the corresponding *p*-value maps, as described by Turley et al.^97^ The boundaries between visual cortical areas 17 and 18, along with the AP0 reference point in the Horsley-Clarke stereotaxic coordinate system, were overlaid on all single-condition functional and *p*-value maps (Figure 8B). Finally, the proportion of the imaging region occupied by significantly activated pixels was calculated to quantitatively assess the spatial characteristics of the cortical responses.

To analyze the temporal dynamics of visual cortical responses evoked by electrical and stimulation (Figures 3 and S6), a rectangular window measuring 1 mm^2^ was first defined. This window was applied in a pixel-wise sliding analysis across the entire field of view for each single-condition functional map, with the mean dR/R signal computed for all pixels within the window at each position. The window exhibiting the minimum mean dR/R value, representing the visually darkest region of the single-condition functional map and corresponding to the locus of maximal response, was selected as the region of interest (ROI), as indicated by the dashed bounding box in Figure 8B. Within the selected ROI, a raw time course of the dR/R signal was generated by averaging pixel intensities across all 720 sequential frames. This time course was then smoothed using a third-order, 0.5 Hz low-pass Butterworth filter to remove high-frequency noise. For each TpES site (across all stimulation intensities) and for TcES, the corresponding time courses were extracted to quantitatively characterize the temporal profile of the evoked cortical responses. Three temporal parameters were systematically quantified for each time course based on the framework established by Sun et al.^11^^,^^43^ Response latency, defined as the interval between the stimulation onset (baseline dR/R map) and the inflection point of the time course, where the inflection point corresponds to the first statistically significant slope change in reflectance.^98^ Peak time, defined as the time at which the maximal response amplitude occurred. Peak duration, defined as the half-peak width, corresponding to the width of the response at half of its maximum amplitude. The definitions of these temporal parameters, derived from a single-condition time course within the selected ROI, are illustrated in Figure 8C.

The amplitude characteristics of visual cortical responses evoked by individual TpES sites and the control TcES were analyzed systematically. The ROI corresponding to the peak response in the single-condition functional map (shown in Figure 8B) was used as a reference and designated ROI 2. An additional ROI was placed anterior to ROI 2 (ROI 1), and three ROIs were positioned posteriorly (ROIs 3-5) along the horizontal axis of the IOS imaging field of view, with a center-to-center spacing of 2-3 mm, as illustrated in the top-right inset of Figure 4. The dR/R values within each ROI were then averaged to quantify variations in response amplitude across different cortical regions under all tested stimulation intensities.

#### Analysis of signal-to-noise ratio (SNR) for visual cortical responses

To assess the robustness of evoked signals beyond statistical significance (*p* < 0.05), we calculated the signal-to-noise ratio (SNR) for both visual grating stimulation and electrical stimulation. First, we computed the time courses of visual cortical responses evoked by low SF (0.14 cpd) and high SF (0.18 cpd) grating stimuli within a single 1 mm^2^ region of interest (ROI) that exhibited the strongest activation in the single-condition functional maps, as illustrated in Figure S6. From these time courses, the peak amplitudes (dR/R) of the intrinsic optical signals (IOS) evoked by visual grating stimuli in Area 18 and Area 17 were calculated to be −0.75 ± 0.05% and −0.71 ± 0.06%, respectively (mean ± SEM, *n* = 11). As shown in Figure 8, among all TpES and TcES conditions tested, the smallest response occurred at 0.3 mA TcES in ROI 5, with a peak amplitude of -0.38 ± 0.04%. Baseline noise was defined as the standard deviation (σ) of dR/R during the 1 s pre-stimulus period. Across typical stimulation-recording blocks (each comprising 128 trials), the baseline noise averaged -0.03% ± 0.003% among 11 animals (mean ± SEM, *n* = 11). Consequently, the SNR for visual stimulation was approximately 24, and even the smallest electrical response yielded an SNR of about 13. These values exceed the baseline noise by more than an order of magnitude, confirming that the statistically significant activation maps reflect robust physiological responses arising from neurovascular coupling in the visual cortex.^99^

Notably, the observed response amplitudes remained substantial and comparable to visually evoked responses under the present isoflurane anesthesia (0.5%–1%), indicating that the anesthetic did not excessively dampen cortical activation or mask genuine differences between stimulation conditions.

#### Computational modeling of retinal electric fields

The spatial distributions of retinal electric fields generated by four independent TpES sites and TcES were simulated using a multi-conductivity human head model reconstructed from MRI data and incorporating anatomically detailed ocular structures (Figure 9A).^80^

The eye model magnified in Figure 9B was constructed in the AC/DC module of COMSOL Multiphysics and validated in our previously published work, its detailed geometric structures were illustrated in Figure S7.^34^^,^^35^^,^^55^^,^^65^ It comprised basic ocular anatomical structures: the cornea, anterior chamber, lens, vitreous body (VB), retina, choroid, and sclera. Adjacent anatomical components were also integrated into the model, including the optic nerve, cerebrospinal fluid (CSF), optic nerve sheath, muscle and fat. The optic nerve was positioned at a 22.5° nasally horizontal tilt relative to the central axis, with a truncated cone-shaped intraocular segment, a cylindrical retrobulbar segment, and coaxial alignment of the optic nerve, CSF, and nerve sheath.

Given that TpES stimulation electrodes were placed on the palpebral surface, a head conductivity model was reconstructed based on real MRI scanning data,^80^ and the detailed eye model was embedded into the head structure according to anatomical landmarks. The detailed procedures are described briefly below:

T1 and T2-weighted MRI data were segmented with the Headreco and CHARM algorithms in SimNIBS software, with high-quality outputs selected to form the initial MASK. The MASK was manually refined in 3D Slicer software, followed by mesh generation in SimNIBS and geometric repair and optimization in Geomagic Wrap software. Finally, tissue models were assembled from outer to inner layers in COMSOL Multiphysics to establish a multi-conductivity head model including skin, muscle, skull, gray matter, white matter, brainstem, and CSF.

Due to the limited spatial resolution of the MRI data (1 mm^3^),^80^ the native ocular structure in the reconstructed head model could not resolve fine tissues including the retina and choroid, and failed to satisfy the requirements of this study for analyzing electric field distributions on the retinal surface. We therefore excised the native ocular structure from the head model, as it only permitted delineation of coarse global contours without intraocular structural detail. The anatomically detailed eye model developed in Figures 9B and S7 was subsequently registered to the native head coordinate system, and integrated into the corresponding anatomical location within the head model via rigid anatomical alignment.

When performing finite element meshing in COMSOL Multiphysics, the custom element size for the electrodes and the retinal layers of interest is set to 2 μm - 0.2 mm. The rest of the eyeball structure is meshed with an extremely refined grid (element size: 7.03 μm - 0.703 mm), while the relevant tissues in the head structure are all meshed with an ultra-refined grid (element size: 52.7 μm - 1.23 mm). This is to ensure sufficient model accuracy during the simulation while meeting the computing power of the hardware equipment.

In the simulation, TpES was delivered through four independent disc electrodes (5 mm in diameter) attached to the superior (CH1), nasal (CH2), inferior (CH3), and temporal (CH4) palpebral skin of the same eye (Figure 9C). Each electrode was positioned approximately 2 cm from the corneal center, consistent with the configuration reported by Lee et al.^49^ TcES was applied using a concentric ring electrode (9.5 mm outer diameter and 8 mm inner diameter) placed directly on the corneal surface of the right eye (Figure 9D), following the setup described by Lu et al.^34^ A separate disc electrode was attached to the neck skin and served as the common return electrode for both stimulation modes, in accordance with protocols described by Gall et al.^81^ The stimulation current applied to each electrode was fixed at 1 mA, and all stimulating and return electrodes were assigned the electrical conductivity of platinum (Pt). The electrical conductivity and geometric parameters of all tissues are derived from published literature and listed in Table S3.^34^^,^^55^^,^^65^^,^^100^^,^^101^

The spatial distribution of electric fields was analyzed within the posterior retina (indicated by the red region in Figure 9E), where effective neuronal activation is essential for restoring visual function.^49^ To facilitate the analysis, a three-dimensional coordinate system was established with the origin (O) located at the center of the retinal surface, and the X, Y, and Z axes aligned with the nasal, anterior, and superior directions, respectively. First, we simulated and calculated the electric field distribution on the retinal surface for all TpES channels and TcES, along with the corresponding XOZ plane and 3D mesh plot. Subsequently, the electric fields generated by all stimulation paradigms were normalized according to their respective maximum and minimum values, and their 80% intensity contours were calculated. These contours were applied to characterize the theoretical activation ranges of TpES and TcES when the maximal electric field intensity on the retinal surface reaches 1.25 times the threshold for neuronal activation.^55^ Three key metrics were derived from this analysis: (1) Maximal central peak intensity of the retinal electric field (Ecp). (2) Central peak localization, quantified by the distance (d) and eccentricity (θ) of the central field peak relative to the origin point. (3) Field distribution profiles, defined as electric field curves with the central peak as the maximum, extending bilaterally along the horizontal and vertical directions.

### Quantification and statistical analysis

All quantitative data derived from IOS imaging of cat’s visual cortex are presented as mean ± standard error of the mean (SEM). A standardized two-step statistical workflow was applied for all group-wise analyses.

1. One-way analysis of variance (ANOVA): A one-way ANOVA was first conducted to assess the overall main effect of a single independent variable on cortical response metrics, including the percentage of the imaging region occupied by significantly activated pixels, temporal parameters derived from each time course, and response amplitudes. The independent variables examined in this study included: (1) stimulation site (four individual TpES sites versus TcES), (2) stimulation current intensity, and (3) cortical ROIs.
2. Post-hoc pairwise comparisons: When a significant main effect was detected by the one-way ANOVA, post-hoc pairwise comparisons were performed using Tukey’s Honestly Significant Difference (HSD) test to identify significant differences between groups, and to control for type I error inflation due to multiple comparisons.

A two-tailed *p*-value <0.05 was considered statistically significant for all tests. Statistical significance is indicated as follows: ∗*p* < 0.05.

### Additional resources

This work has not generated or contributed to a new website/forum or has not been part of a clinical trial.

## References

[1] Li J., Zhou W., Liang L., Li Y., Xu K., Li X., Huang Z., Jin Y. (2024). Noninvasive electrical stimulation as a neuroprotective strategy in retinal diseases: a systematic review of preclinical studies. J. Transl. Med..

[2] Morimoto T. (2025). Transcorneal electrical stimulation: impact on healthcare and future potential. Front. Cell Dev. Biol..

[3] Demir M.N., Acar U., Sobacı G., Göksülük D. (2022). Outcomes of transcorneal electrical stimulation therapy in the early stages of retinitis pigmentosa. Turk. J. Med. Sci..

[4] Meral N., Zabek O., Camenzind Zuche H., Müller U., Prétot D., Rickmann A., Scholl H.P.N., Della Volpe Waizel M. (2022). Metabolic Long-Term Monitoring of Transcorneal Electrical Stimulation in Retinitis Pigmentosa. Ophthalmic Res..

[5] Stett A., Schatz A., Gekeler F., Franklin J. (2023). Transcorneal Electrical Stimulation Dose-Dependently Slows the Visual Field Loss in Retinitis Pigmentosa. Transl. Vis. Sci. Technol..

[6] Tao Y., Chen T., Liu Z.Y., Wang L.Q., Xu W.W., Qin L.M., Peng G.H., Yi-Fei H. (2016). Topographic Quantification of the Transcorneal Electrical Stimulation (TES)-Induced Protective Effects on N-Methyl-N-Nitrosourea-Treated Retinas. Investig. Ophthalmol. Vis. Sci..

[7] Jassim A.H., Cavanaugh M., Shah J.S., Willits R., Inman D.M. (2021). Transcorneal Electrical Stimulation Reduces Neurodegenerative Process in a Mouse Model of Glaucoma. Ann. Biomed. Eng..

[8] Tew B.Y., Gooden G.C., Lo P.A., Pollalis D., Ebright B., Kalfa A.J., Gonzalez-Calle A., Thomas B., Buckley D.N., Simon T. (2024). Transcorneal electrical stimulation restores DNA methylation changes in retinal degeneration. Front. Mol. Neurosci..

[9] Ma Z., Cao P., Sun P., Li L., Lu Y., Yan Y., Chen Y., Chai X. (2014). Optical imaging of visual cortical responses evoked by transcorneal electrical stimulation with different parameters. Investig. Ophthalmol. Vis. Sci..

[10] Morimoto T., Kanda H., Miyoshi T., Hirohara Y., Mihashi T., Kitaguchi Y., Nishida K., Fujikado T. (2014). Characteristics of retinal reflectance changes induced by transcorneal electrical stimulation in cat eyes. PLoS One.

[11] Sun P., Li H., Lu Z., Su X., Ma Z., Chen J., Li L., Zhou C., Chen Y., Chai X. (2018). Comparison of cortical responses to the activation of retina by visual stimulation and transcorneal electrical stimulation. Brain Stimul..

[12] Xie J., Wang G.J., Yow L., Humayun M.S., Weiland J.D., Cela C.J., Jadvar H., Lazzi G., Dhrami-Gavazi E., Tsang S.H. (2012). Preservation of retinotopic map in retinal degeneration. Exp. Eye Res..

[13] Wagner S., Süer E., Sigdel B., Zrenner E., Strasser T. (2023). Monocular transcorneal electrical stimulation induces ciliary muscle thickening in contralateral eye. Exp. Eye Res..

[14] Naycheva L., Schatz A., Willmann G., Bartz-Schmidt K.U., Zrenner E., Röck T., Gekeler F. (2013). Transcorneal electrical stimulation in patients with retinal artery occlusion: a prospective, randomized, sham-controlled pilot study. Ophthalmol. Ther..

[15] Schatz A., Pach J., Gosheva M., Naycheva L., Willmann G., Wilhelm B., Peters T., Bartz-Schmidt K.U., Zrenner E., Messias A., Gekeler F. (2017). Transcorneal Electrical Stimulation for Patients With Retinitis Pigmentosa: A Prospective, Randomized, Sham-Controlled Follow-up Study Over 1 Year. Investig. Ophthalmol. Vis. Sci..

[16] Liu J., Tong K., Lin Y., Lee V.W.H., So K.F., Shih K.C., Lai J.S.M., Chiu K. (2021). Effectiveness of Microcurrent Stimulation in Preserving Retinal Function of Blind Leading Retinal Degeneration and Optic Neuropathy: A Systematic Review. Neuromodulation.

[17] Granata G., Falsini B. (2023). Preliminary Results of Transorbital Alternating Current Stimulation in Chronic Low Vision: Correlation of Clinical and Neurophysiological Results. Neuromodulation.

[18] Anastassiou G., Schneegans A.L., Selbach M., Kremmer S. (2013). Transpalpebral electrotherapy for dry age-related macular degeneration (AMD): an exploratory trial. Restor. Neurol. Neurosci..

[19] Colombo L., Caretti A., Dei Cas M., Luciano F., Romano D., Paroni R., Patelli F., Ghidoni R., Rossetti L. (2021). Vitreous composition modification after transpalpebral electrical stimulation of the eye: Biochemical analysis. Exp. Eye Res..

[20] Parkinson K.M., Sayre E.C., Tobe S.W. (2023). Evaluation of visual acuity in dry AMD patients after microcurrent electrical stimulation. Int. J. Retina Vitreous.

[21] Martella S., Ferri P., Colombo L., Torregrossa G., Mocciardini E., Baldesi J., Quisisana C., Rossetti L. (2025). Restoring visual function in NAION by means of transpalpebral electrical stimulation: A case report. Eur. J. Ophthalmol..

[22] Yang M., Lennikov A., Chang K., Ashok A., Lee C., Cho K.S., Utheim T.P., Dartt D.A., Chen D.F. (2022). Transcorneal but not transpalpebral electrical stimulation disrupts mucin homeostasis of the ocular surface. BMC Ophthalmol..

[23] Eghrari A.O., Riazuddin S.A., Gottsch J.D. (2015). Overview of the Cornea: Structure, Function, and Development. Prog. Mol. Biol. Transl. Sci..

[24] Kumar A., Yun H., Funderburgh M.L., Du Y. (2022). Regenerative therapy for the Cornea. Prog. Retin. Eye Res..

[25] Koch E., Jin J., Alonso J.M., Zaidi Q. (2016). Functional implications of orientation maps in primary visual cortex. Nat. Commun..

[26] Zhou W., Huang Z., Xu K., Li Y., Li X., Li J., Jin Y., Snellingen T., Liang L. (2024). Transpalpebral electrical stimulation for the treatment of retinitis pigmentosa: study protocol for a series of N-of-1 single-blind, randomized controlled trial. Trials.

[27] Schittkowski M., Pohlner J., Mercieca K., Grohmann C., Kröger L., Prokosch V., Lorenz K., Beck A., Haueisen J., Hunold A. (2025). Vision Restoration through transorbital electrical stimulation in Optic Neuropathy in patients with significant optic atrophy due to primary open-angle glaucoma-a randomised, controlled, double-blind, multicentre clinical trial: the VIRON study protocol. BMJ Open.

[28] Garrett M.E., Nauhaus I., Marshel J.H., Callaway E.M. (2014). Topography and areal organization of mouse visual cortex. J. Neurosci..

[29] Najafian S., Jin J., Alonso J.M. (2019). Diversity of Ocular Dominance Patterns in Visual Cortex Originates from Variations in Local Cortical Retinotopy. J. Neurosci..

[30] Broderick W.F., Simoncelli E.P., Winawer J. (2022). Mapping spatial frequency preferences across human primary visual cortex. J. Vis..

[31] Williams B., Del Rosario J., Muzzu T., Peelman K., Coletta S., Bichler E.K., Speed A., Meyer-Baese L., Saleem A.B., Haider B. (2021). Spatial modulation of dark versus bright stimulus responses in the mouse visual system. Curr. Biol..

[32] Wang H., Dey O., Lagos W.N., Behnam N., Callaway E.M., Stafford B.K. (2024). Parallel pathways carrying direction-and orientation-selective retinal signals to layer 4 of the mouse visual cortex. Cell Rep..

[33] Rodríguez Deliz C.L., Lee G.M., Bushnell B.N., Majaj N.J., Movshon J.A., Kiorpes L. (2025). Neural Sensitivity to Radial Frequency Patterns in the Visual Cortex of Developing Macaques. J. Neurosci..

[34] Lu Z., Zhou M., Guo T., Liang J., Wu W., Gao Q., Li L., Li H., Chai X. (2022). An in-silico analysis of retinal electric field distribution induced by different electrode design of trans-corneal electrical stimulation. J. Neural. Eng..

[35] Song X., Guo T., Ma S., Zhou F., Tian J., Liu Z., Liu J., Li H., Chen Y., Chai X., Li L. (2025). Spatially Selective Retinal Ganglion Cell Activation Using Low Invasive Extraocular Temporal Interference Stimulation. Int. J. Neural Syst..

[36] Tusa R.J., Rosenquist A.C., Palmer L.A. (1979). Retinotopic organization of areas 18 and 19 in the cat. J. Comp. Neurol..

[37] Tusa R.J., Palmer L.A., Rosenquist A.C. (1978). The retinotopic organization of area 17 (striate cortex) in the cat. J. Comp. Neurol..

[38] Burke W., Dreher B., Wang C. (1998). Selective block of conduction in Y optic nerve fibres: significance for the concept of parallel processing. Eur. J. Neurosci..

[39] Troy J.B., Shou T. (2002). The receptive fields of cat retinal ganglion cells in physiological and pathological states: where we are after half a century of research. Prog. Retin. Eye Res..

[40] Narfström K., Deckman K.H., Menotti-Raymond M. (2013). Cats: a gold mine for ophthalmology. Annu. Rev. Anim. Biosci..

[41] Lu Y., Yan Y., Chai X., Ren Q., Chen Y., Li L. (2013). Electrical stimulation with a penetrating optic nerve electrode array elicits visuotopic cortical responses in cats. J. Neural. Eng..

[42] Ma Z., Cao P., Sun P., Zhao L., Li L., Tong S., Lu Y., Yan Y., Chen Y., Chai X. (2016). Inverted optical intrinsic response accompanied by decreased cerebral blood flow are related to both neuronal inhibition and excitation. Sci. Rep..

[43] Su X., Zhou M., Di L., Chen J., Zhai Z., Liang J., Li L., Li H., Chai X. (2022). The visual cortical responses to sinusoidal transcorneal electrical stimulation. Brain Res..

[44] Pi S., Wang B., Gao M., Cepurna W., Lozano D.C., Morrison J.C., Jia Y. (2023). Longitudinal Observation of Retinal Response to Optic Nerve Transection in Rats Using Visible Light Optical Coherence Tomography. Investig. Ophthalmol. Vis. Sci..

[45] Bjånes D.A., Kellis S., Nickl R., Baker B., Aflalo T., Bashford L., Chivukula S., Fifer M.S., Osborn L.E., Christie B. (2025). Quantifying physical degradation alongside recording and stimulation performance of 980 intracortical microelectrodes chronically implanted in three humans for 956-2130 days. Acta Biomater..

[46] Shmuel A., Augath M., Oeltermann A., Logothetis N.K. (2006). Negative functional MRI response correlates with decreases in neuronal activity in monkey visual area V1. Nat. Neurosci..

[47] Ma Z., Cao P., Sun P., Lu Z., Li L., Chen Y., Chai X. (2017). Negative hemodynamic response without neuronal inhibition investigated by combining optical imaging and electrophysiological recording. Neurosci. Lett..

[48] Haberbosch L., Datta A., Thomas C., Jooß A., Köhn A., Rönnefarth M., Scholz M., Brandt S.A., Schmidt S. (2019). Safety Aspects, Tolerability and Modeling of Retinofugal Alternating Current Stimulation. Front. Neurosci..

[49] Lee S., Park J., Kwon J., Kim D.H., Im C.H. (2021). Multi-channel transorbital electrical stimulation for effective stimulation of posterior retina. Sci. Rep..

[50] Bouisset N., Carvallo A., Laporte M., Legros A. (2025). Human achromatic flickers and phosphenes thresholds under extremely low frequency electric stimulations. Sci. Rep..

[51] Reato D., Rahman A., Bikson M., Parra L.C. (2010). Low-intensity electrical stimulation affects network dynamics by modulating population rate and spike timing. J. Neurosci..

[52] Sabel B.A., Hamid A.I.A., Borrmann C., Speck O., Antal A. (2020). Transorbital alternating current stimulation modifies BOLD activity in healthy subjects and in a stroke patient with hemianopia: A 7 Tesla fMRI feasibility study. Int. J. Psychophysiol..

[53] Hunold A., Ortega D., Freitag S., Link D., Antal A., Klee S., Haueisen J. (2025). Retinal Phosphenes Induced by Transorbital Electrical Stimulation: Influence of Light Adaptation, Electrode Montage, and View Direction. Life.

[54] Kim H.M., Park Y.J., Park K.H., Woo S.J. (2019). Visual field defects and changes in central retinal artery occlusion. PLoS One.

[55] Su X., Guo J., Zhou M., Chen J., Li L., Chen Y., Sui X., Li H., Chai X. (2021). Computational Modeling of Spatially Selective Retinal Stimulation With Temporally Interfering Electric Fields. IEEE Trans. Neural Syst. Rehabil. Eng..

[56] Purves D. (2025). Understanding Visual Perception. J. Cognit. Neurosci..

[57] Lyu Q., Lu Z., Li H., Qiu S., Guo J., Sui X., Sun P., Li L., Chai X., Lovell N.H. (2020). A Three-Dimensional Microelectrode Array to Generate Virtual Electrodes for Epiretinal Prosthesis Based on a Modeling Study. Int. J. Neural Syst..

[58] Choi J.H., Moon J., Park Y.H., Eom K. (2024). Computational analysis of electrode structure and configuration for efficient and localized neural stimulation. Biomed. Eng. Lett..

[59] Allison-Walker T., Hagan M.A., Price N.S.C., Wong Y.T. (2021). Microstimulation-evoked neural responses in visual cortex are depth dependent. Brain Stimul..

[60] Boycott B.B., Kolb H. (1973). The connections between bipolar cells and photoreceptors in the retina of the domestic cat. J. Comp. Neurol..

[61] Picaud S., Dalkara D., Marazova K., Goureau O., Roska B., Sahel J.A. (2019). The primate model for understanding and restoring vision. Proc. Natl. Acad. Sci. USA.

[62] Opitz A., Falchier A., Yan C.G., Yeagle E.M., Linn G.S., Megevand P., Thielscher A., Deborah A R., Milham M.P., Mehta A.D., Schroeder C.E. (2016). Spatiotemporal structure of intracranial electric fields induced by transcranial electric stimulation in humans and nonhuman primates. Sci. Rep..

[63] Alekseichuk I., Mantell K., Shirinpour S., Opitz A. (2019). Comparative modeling of transcranial magnetic and electric stimulation in mouse, monkey, and human. Neuroimage.

[64] Schramm U., Unger K., Keeler C. (1994). Functional morphology of the nictitating membrane in the domestic cat. Ann. Anat..

[65] Zhou M., Su X., Guo T., Meng T., Wu W., Di L., Li L., Li H., Chai X. (2024). Optic nerve-mediated modulation of temporally interfering electric fields for potential targeted retinal disease therapy: a computational modeling study. Front. Neurosci..

[66] Yin R., Xu Z., Mei M., Chen Z., Wang K., Liu Y., Tang T., Priydarshi M.K., Meng X., Zhao S. (2018). Soft transparent graphene contact lens electrodes for conformal full-cornea recording of electroretinogram. Nat. Commun..

[67] Shepherd R.K., Carter P.M., Dalrymple A.N., Enke Y.L., Wise A.K., Nguyen T., Firth J., Thompson A., Fallon J.B. (2021). Platinum dissolution and tissue response following long-term electrical stimulation at high charge densities. J. Neural. Eng..

[68] Yeung V., Boychev N., Farhat W., Ntentakis D.P., Hutcheon A.E.K., Ross A.E., Ciolino J.B. (2022). Extracellular Vesicles in Corneal Fibrosis/Scarring. Int. J. Mol. Sci..

[69] Wilson S.E. (2023). Topical Losartan: Practical Guidance for Clinical Trials in the Prevention and Treatment of Corneal Scarring Fibrosis and Other Eye Diseases and Disorders. J. Ocul. Pharmacol. Ther..

[70] Mathias R.T., Rae J.L., Eisenberg R.S. (1979). Electrical properties of structural components of the crystalline lens. Biophys. J..

[71] Lindenblatt G., Silny J. (2002). Electrical phosphenes: on the influence of conductivity inhomogeneities and small-scale structures of the orbita on the current density threshold of excitation. Med. Biol. Eng. Comput..

[72] Ramezanpour H., Kehoe D.H., Perry C.J., Fallah M. (2026). Visual attention in peripersonal space is dependent on differential modulation of V2 feature selectivity by hand vision and proprioception. Curr. Biol..

[73] Lee H.J., Shin H.K., Shin Y.I., Kim J.H., Choi B.T. (2026). The Fundamental Mechanism of Transcranial Electrical Stimulation in Post-Stroke Rehabilitation. Front. Biosci..

[74] Huang Y., Liu A.A., Lafon B., Friedman D., Dayan M., Wang X., Bikson M., Doyle W.K., Devinsky O., Parra L.C. (2017). Measurements and models of electric fields in the in vivo human brain during transcranial electric stimulation. eLife.

[75] Kasten F.H., Duecker K., Maack M.C., Meiser A., Herrmann C.S. (2019). Integrating electric field modeling and neuroimaging to explain inter-individual variability of tACS effects. Nat. Commun..

[76] Boycott B.B., Wässle H. (1974). The morphological types of ganglion cells of the domestic cat's retina. J. Physiol..

[77] Cleland B.G., Levick W.R. (1974). Brisk and sluggish concentrically organized ganglion cells in the cat's retina. J. Physiol..

[78] Yaghmazadeh O., Alon L., Arefin T.M., Ben Youss Z., Zhang J., Buzsáki G. (2026). Non-invasive modulation of brain activity and behavior by transcranial radio frequency stimulation. Brain Stimul..

[79] Sun P., Li Q., Li H., Di L., Su X., Chen J., Zheng H., Chen Y., Zhou C., Chai X. (2019). Depth-Resolved Physiological Response of Retina to Transcorneal Electrical Stimulation Measured With Optical Coherence Tomography. IEEE Trans. Neural Syst. Rehabil. Eng..

[80] Cvetković M., Dodig H., Poljak D. (2017). A Study on the Use of Compound and Extracted Models in the High Frequency Electromagnetic Exposure Assessment. Math. Probl Eng..

[81] Gall C., Schmidt S., Schittkowski M.P., Antal A., Ambrus G.G., Paulus W., Dannhauer M., Michalik R., Mante A., Bola M. (2016). Alternating Current Stimulation for Vision Restoration after Optic Nerve Damage: A Randomized Clinical Trial. PLoS One.

[82] Chen D., Greenstein V.C., Brodie S.E. (2022). Qualitative and quantitative comparison of ERGs with contact lens and adhesive skin electrodes. Doc. Ophthalmol..

[83] Eckermann T., Hoffmann M.B., Al-Nosairy K.O. (2023). Comparison of DTL and gold cup skin electrodes for recordings of the multifocal electroretinogram. Doc. Ophthalmol..

[84] Sinim Kahraman N., Oner A. (2020). Effect of Transcorneal Electrical Stimulation on Patients with Retinitis Pigmentosa. J. Ocul. Pharmacol. Therapeut..

[85] Wagner S.K., Jolly J.K., Pefkianaki M., Gekeler F., Webster A.R., Downes S.M., Maclaren R.E. (2017). Transcorneal electrical stimulation for the treatment of retinitis pigmentosa: results from the TESOLAUK trial. BMJ Open Ophthalmol..

[86] Ly K., Italiano M.L., Shivdasani M.N., Tsai D., Zhang J.Y., Jiang C., Lovell N.H., Dokos S., Guo T. (2025). Virtual human retina: Simulating neural signalling, degeneration, and responses to electrical stimulation. Brain Stimul..

[87] Laakso I., Tanaka S., Koyama S., De Santis V., Hirata A. (2015). Inter-subject Variability in Electric Fields of Motor Cortical tDCS. Brain Stimul..

[88] Pu Y., Liu Z., Ye L., Xia Y., Chen X., Wang K., Pierscionek B.K. (2023). The major influence of anterior and equatorial zonular fibres on the far-to-near accommodation revealed by a 3D pre-stressed model of the anterior eye. Comput. Methods Programs Biomed..

[89] Kim U.S., Mahroo O.A., Mollon J.D., Yu-Wai-Man P. (2021). Retinal Ganglion Cells-Diversity of Cell Types and Clinical Relevance. Front. Neurol..

[90] Prakash N., Uhlemann F., Sheth S.A., Bookheimer S., Martin N., Toga A.W. (2009). Current trends in intraoperative optical imaging for functional brain mapping and delineation of lesions of language cortex. Neuroimage.

[91] Zepeda A., Arias C., Sengpiel F. (2004). Optical imaging of intrinsic signals: recent developments in the methodology and its applications. J. Neurosci. Methods.

[92] Fernald R., Chase R. (1971). An improved method for plotting retinal landmarks and focusing the eyes. Vis. Res..

[93] Issa N.P., Trepel C., Stryker M.P. (2000). Spatial frequency maps in cat visual cortex. J. Neurosci..

[94] Huang L., Chen X., Shou T. (2004). Spatial frequency-dependent feedback of visual cortical area 21a modulating functional orientation column maps in areas 17 and 18 of the cat. Brain Res..

[95] Ananyev S., Sakun I., Lyakhovetskii V., Grishin A., Moshonkina T., Gerasimenko Y. (2026). Modulation of Forward Propulsion and Foot Dorsiflexion by Spinal and Muscular Stimulation During Human Stepping. Life.

[96] de Rossi F., Guidobaldi M., Turco S., Amore F. (2020). Transorbital electrical stimulation in retinitis pigmentosa. Better results joining visual pattern stimulation?. Brain Stimul..

[97] Turley J.A., Zalewska K., Nilsson M., Walker F.R., Johnson S.J. (2017). An analysis of signal processing algorithm performance for cortical intrinsic optical signal imaging and strategies for algorithm selection. Sci. Rep..

[98] Sirotin Y.B., Hillman E.M.C., Bordier C., Das A. (2009). Spatiotemporal precision and hemodynamic mechanism of optical point spreads in alert primates. Proc. Natl. Acad. Sci. USA.

[99] Thapa D., Wang B., Lu Y., Son T., Yao X. (2017). Enhancement of intrinsic optical signal recording with split spectrum optical coherence tomography. J. Mod. Opt..

[100] Norman R.E., Flanagan J.G., Sigal I.A., Rausch S.M.K., Tertinegg I., Ethier C.R. (2011). Finite element modeling of the human sclera: influence on optic nerve head biomechanics and connections with glaucoma. Exp. Eye Res..

[101] Tsukitome H., Hatsukawa Y., Morimitsu T., Yagasaki T., Kondo M. (2015). Changes in angle of optic nerve and angle of ocular orbit with increasing age in Japanese children. Br. J. Ophthalmol..

