# Supplementary material for: Spatiotemporal characteristics of visual cortical responses to transpalpebral electrical stimulation
Source: iScience. 2026 Jul 10;29(8):116705. doi: 10.1016/j.isci.2026.116705 (PMC13380434; doi:10.1016/j.isci.2026.116705)
Supplement: Document S1. Figures S1–S7 and Tables S1–S3 [file mmc1.pdf]

iScience, Volume *29*

Supplemental information

Spatiotemporal characteristics of visual cortical responses to transpalpebral electrical stimulation

Meixuan Zhou, Yiheng Xu, Tianyue Meng, Tianruo Guo, Yanyang Zhang, Liqing Di, Liming Li, Heng Li, and Xinyu Chai

Figure S1 Temporal sequence of dR/R maps evoked by TpES and TcES at 3.6 mA. Each map was time-binned at 1 s resolution, with 40 frames in total. Dotted box: ROI in the activated region for temporal analysis. Vertical lines: AP0 reference (Horsley-Clarke coordinate system); oblique lines: estimated boundary between Areas 17 and 18. Black vertical bar: stimulation duration. Related to Figure 3.

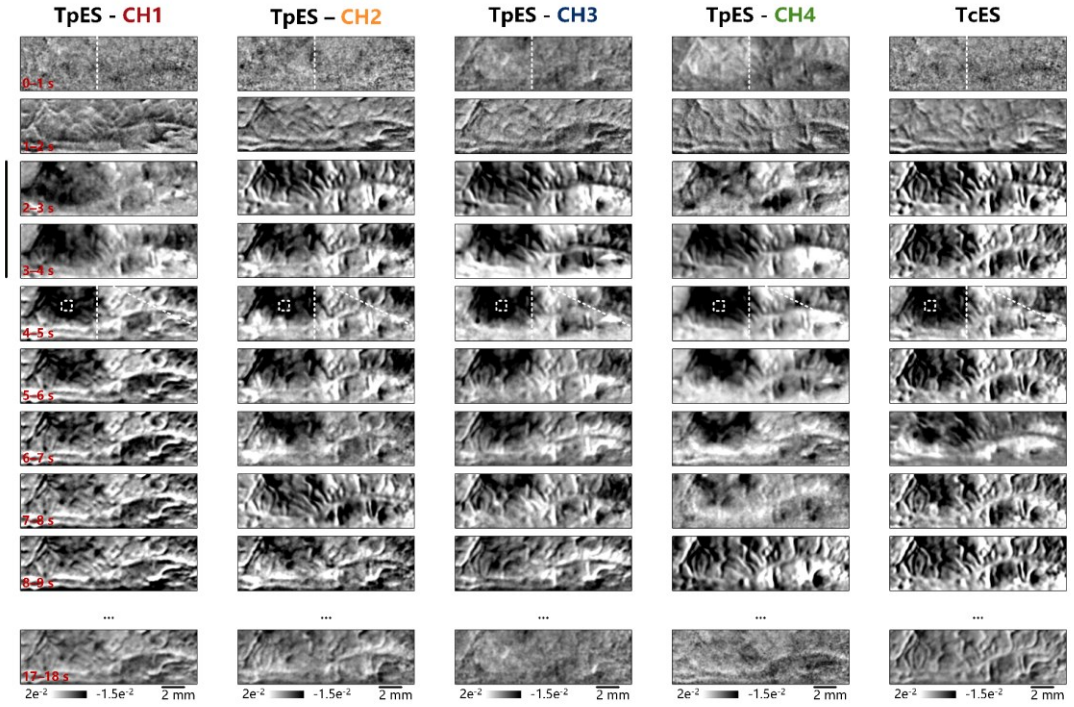

Figure S2 Spatial distribution of current streams from four independent TpES channels and TcES in the multi-conductivity head model. (A) Sagittal section. (B) Horizontal section. Dark curves with arrows: Distributions of current streams in the head model. Red arrows: Current streams circumventing the eyeball to reach the corresponding retinal edge at the TpES stimulating electrode site. Color bar indicates the absolute electric field amplitudes on the retinal surface. Related to Figure 6.

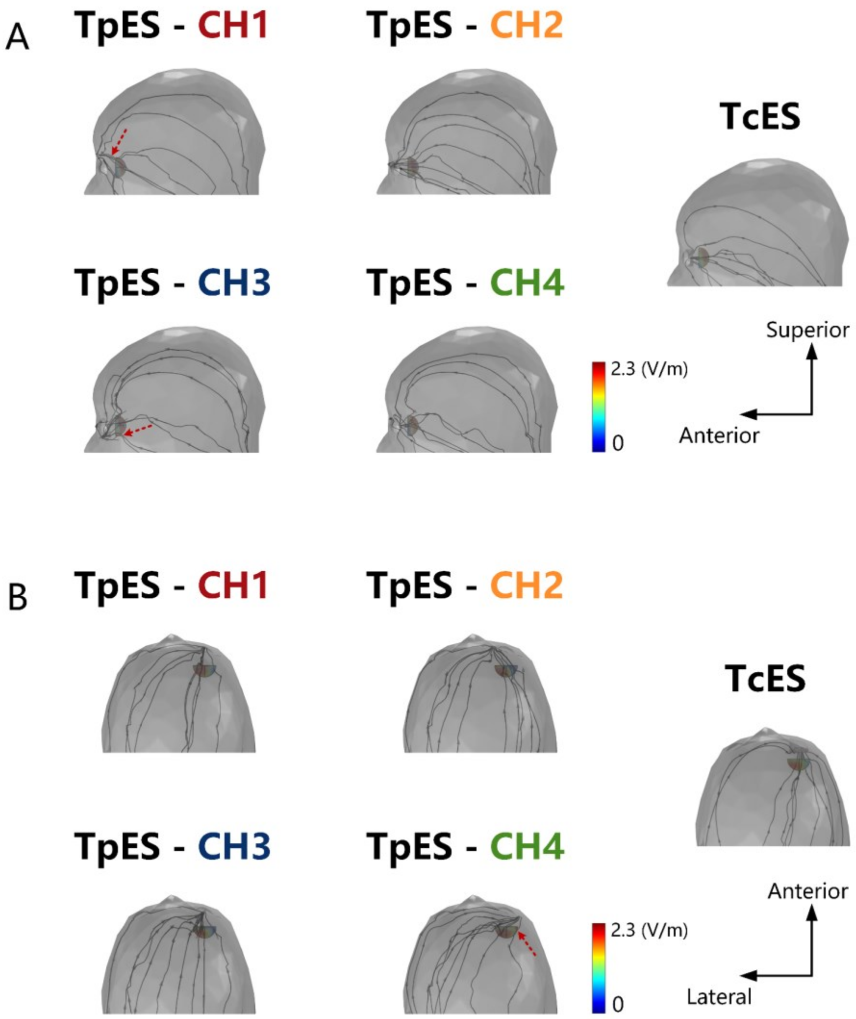

Figure S3 The electric field distribution within the whole brain generated by TpES-CH1 to CH4 and TcES. Dashed box: peri-visual cortex region. Color bar indicates the absolute electric field amplitudes in the whole brain. Related to Figure 6.

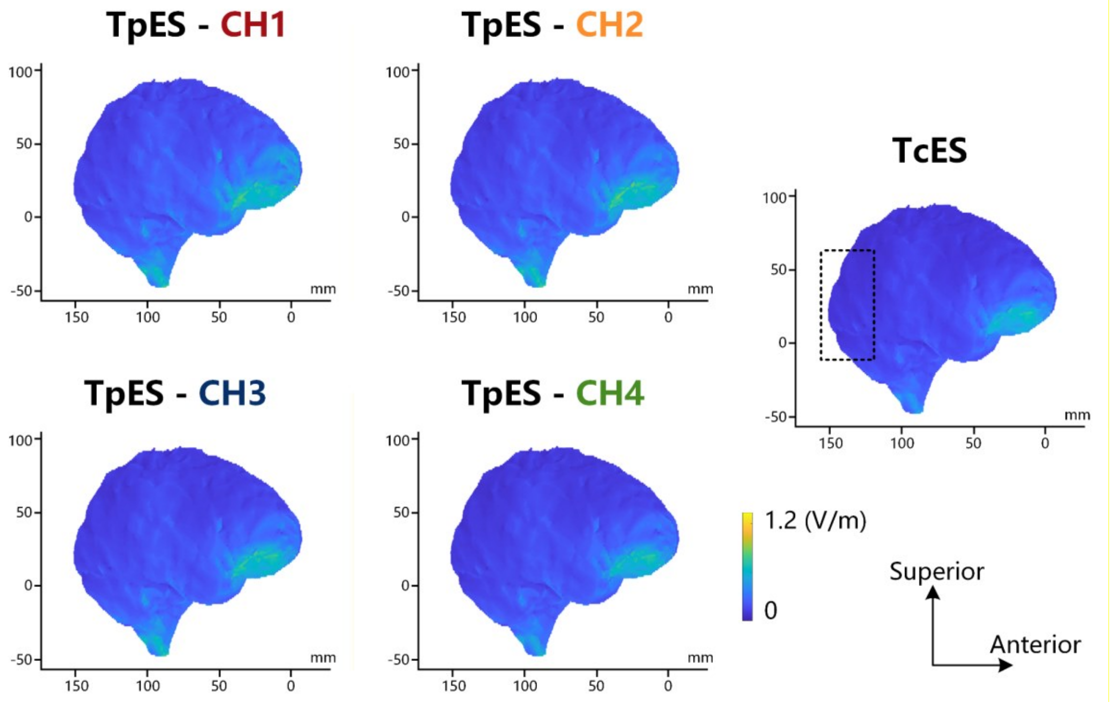

Figure S4 Flow chart illustrating the IOS data analysis process by visual stimulation. The vertical dashed line indicates the Horsley-Clarke coordinate AP0 for one animal, and the oblique line demarcates the boundary between Areas 17 and 18. The middle panel shows sinusoidal drifting gratings used for visual stimulation at low and high spatial frequencies (SF), with horizontal (0°) and vertical (90°) orientations. Related to STAR Methods.

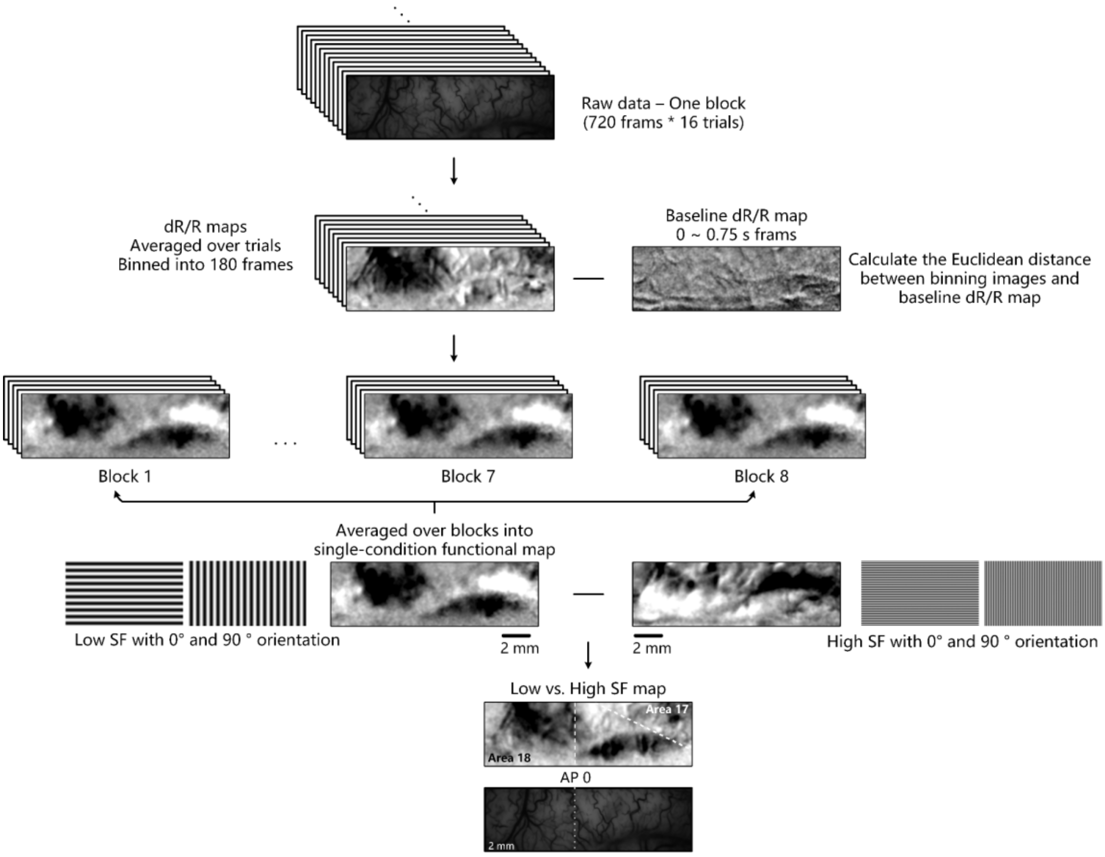

Figure S5 Flow chart explaining the IOS data analysis process evoked by TpES and TcES. Related to STAR Methods.

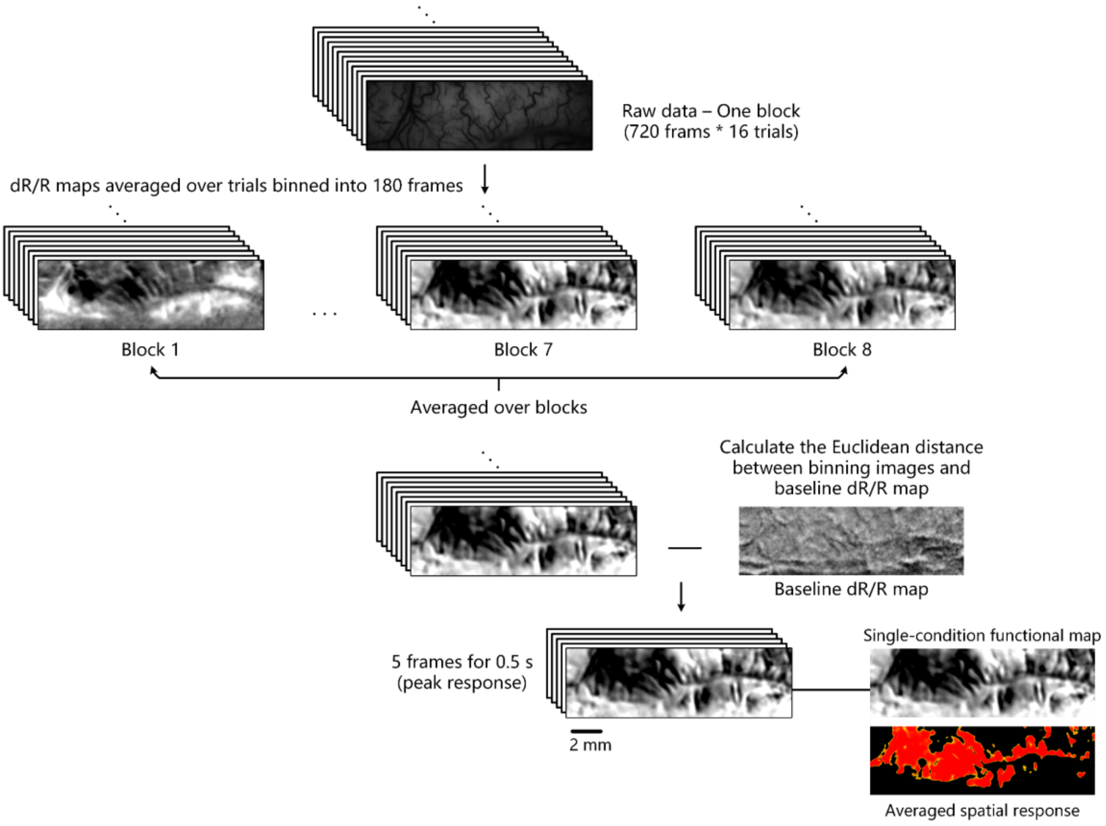

Figure S6 Time courses of visual cortical responses evoked by grating stimulation. Responses were derived from a 1 mm² ROI showing the strongest activation in single-condition functional maps. Solid lines in lower panels indicate the duration of visual grating stimulation. (A) Low SF, 014 cpd. (B) High SF, 0.58 cpd. Related to STAR Methods.

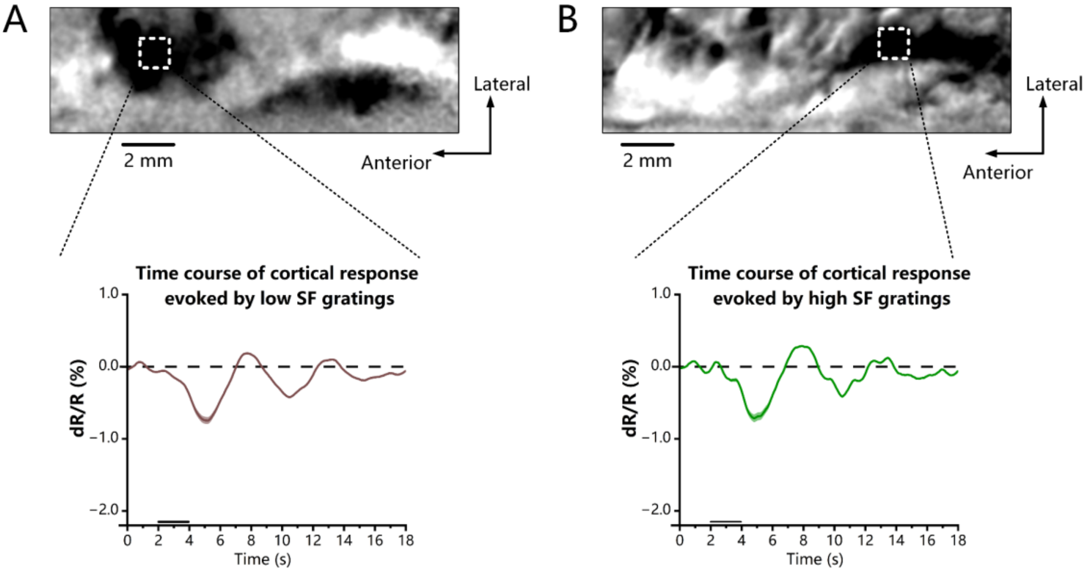

Figure S7 Schematic of structural parameters for the detailed eye model with optic nerve and related structures. (A) Transverse cross-section. (B) Sagitta cross-section. Related to STAR Methods.

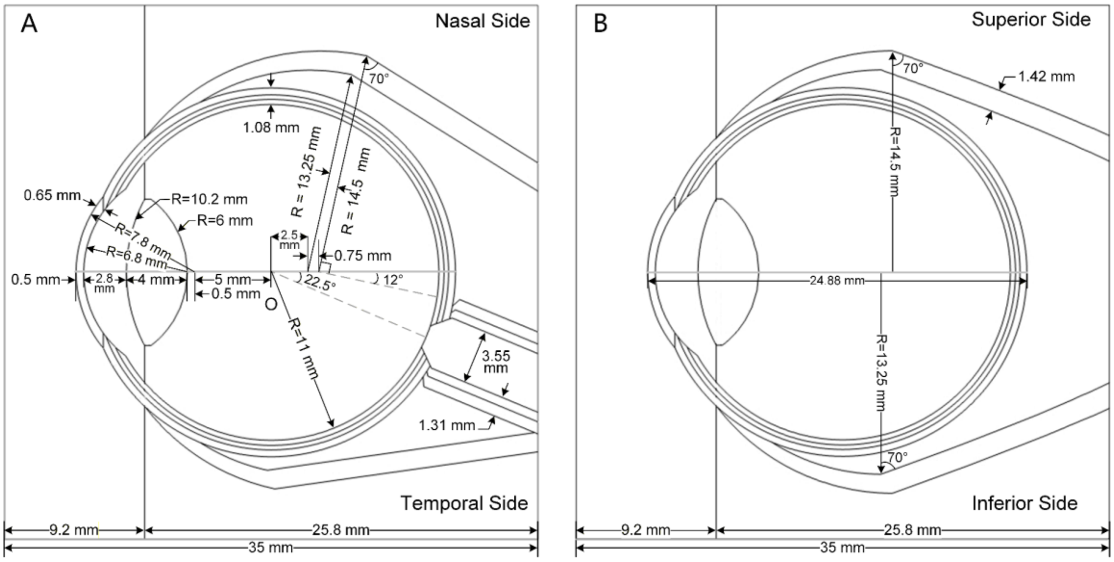

Table S1 Percentages of significantly activated cortical area (mean ± SEM, %) in visual cortical Areas 18 and 17 across four TpES stimulation sites and TcES at all tested current intensities (n = 11 per group). Separate one-way ANOVAs were conducted for each current intensity to compare group differences, with corresponding F and exact *p* values listed in the two rightmost columns. Related to Figure 2.

**Areas**	I (mA)	Averaged activated pixel ratios (%)					F (4, 40)	p
		TpES - CH1	TpES - CH2	TpES - CH3	TpES - CH4	TcES		
**18**	0.3	28.38 ± 2.65	25.27 ± 3.38	28.66 ± 2.95	28.61 ± 1.99	29.19 ± 3.23	0.278	**0.891**
	0.6	33.29 ± 2.68	32.84 ± 2.88	32.27 ± 3.92	34.01 ± 4.27	33.38 ± 3.18	0.030	**0.998**
	1.2	38.67 ± 4.77	40.41 ± 3.74	41.53 ± 4.69	43.06 ± 4.81	42.78 ± 4.71	0.139	**0.967**
	2.4	48.28 ± 4.95	44.45 ± 4.97	52.55 ± 5.32	47.35 ± 5.19	46.84 ± 5.19	0.334	**0.854**
	3.6	57.78 ± 5.94	54.09 ± 4.66	56.32 ± 5.83	55.58 ± 6.23	56.59 ± 6.04	0.056	**0.994**
	4.8	62.58 ± 5.66	59.91 ± 6.87	61.37 ± 6.23	64.24 ± 6.63	62.36 ± 7.01	0.060	**0.993**
**17**	0.3	0.93 ± 0.13	0.83 ± 0.10	1.17 ± 0.11	0.98 ± 0.10	0.98 ± 0.11	1.085	**0.377**
	0.6	1.40 ± 0.19	1.48 ± 0.23	2.13 ± 0.33	2.24 ± 0.33	2.06 ± 0.29	1.632	**0.185**
	1.2	3.17 ± 0.30	3.13 ± 0.35	3.88 ± 0.42	3.99 ± 0.49	3.99 ± 0.62	0.990	**0.429**
	2.4	6.89 ± 0.98	6.13 ± 0.59	6.04 ± 0.60	5.93 ± 0.65	6.81 ± 0.82	0.367	**0.831**
	3.6	10.80 ± 1.56	10.69 ± 1.20	10.22 ± 1.22	12.50 ± 1.78	10.73 ± 1.54	0.353	**0.841**
	4.8	15.01 ± 2.03	13.78 ± 1.74	14.98 ± 1.86	16.42 ± 1.70	14.22 ± 1.56	0.317	**0.866**

All 11 animals received electrical stimulation at 5 sites and 6 current intensities, resulting in a one-way repeated-measures ANOVA with degrees of freedom $F(4, 40)$. $p < 0.05$ indicates a significant main effect.

Table S2 Electric field intensities in different regions and electrode potentials. Maximal electric field intensities at the superonasal central peak (Ecp), central retina and peripheral retinal edge under each stimulating paradigms, as well as corresponding electrode potentials, and maximum electric field intensities in the whole brain and peri-visual cortex. Related to Figure 6.

Regions	TpES-CH1	TpES-CH2	TpES-CH3	TpES-CH4	TcES
Ecp (V/m)	2.18	2.14	2.16	2.24	2.27
Central retina (V/m)	1.69	1.63	1.65	1.76	1.74
Peripheral retinal edge (V/m)	2.04	1.88	2.03	2.11	1.63
Electrode potential (V)	0.90	0.88	0.88	0.88	0.68
Whole brain (V/m)	1.09	1.11	1.01	0.92	0.83
Peri-visual cortex (V/m)	0.24	0.23	0.21	0.18	0.15

Table S3 Electrical conductivities and anatomical geometric parameters of the multi-conductivity human head model with anatomically detailed eye structures. Related to Figure 9.

Tissue/Body fluid/Electrode	Conductivity (S/m)	Thickness (mm)
Skin	0.43	-
Muscle (head)	0.32	-
Fat (head)	0.028	
Skull	0.015	-
Gray matter	0.32	-
White matter	0.15	-
Brainstem	0.47	-
Cornea	0.422	0.5
Anterior chamber	1.5	2.8
Lens	0.322	4.0
Vitreous body	1.5	22*
Retina	0.5028	0.33
Muscle (eye)	0.32	1.42
Fat (eye)	0.028	14.5
Choroid	0.2779	0.45*
Sclera	0.5028	0.63
Optic nerve	axial: 0.5<br>sectional: 0.08	3.55*
CSF	2	0.58
Optic nerve sheath	0.006	0.73
Electrode (Pt)	8.9×10^{6}	-

* The thickness of VB and optic nerve represent maximum diameters.
